# How doctors diagnose diseases and prescribe treatments: an fMRI study of diagnostic salience

**DOI:** 10.1038/s41598-017-01482-0

**Published:** 2017-05-02

**Authors:** Marcio Melo, Gustavo D. F. Gusso, Marcelo Levites, Edson Amaro Jr., Eduardo Massad, Paulo A. Lotufo, Peter Zeidman, Cathy J. Price, Karl J. Friston

**Affiliations:** 1 Laboratory of Medical Investigations, LIM-01, Faculty of Medicine of the University of São Paulo, Av. Dr. Arnaldo 455, São Paulo, 01246-904 Brazil; 2 Albert Einstein Israelite Hospital, IIEP, Av. Albert Einstein 627, São Paulo, 05652-900 Brazil; 3 Department of Internal Medicine, Faculty of Medicine of the University of São Paulo, Av. Dr. Eneas de Carvalho Aguiar 155, São Paulo, 05403-000 Brazil; 4 Department of Radiology, Faculty of Medicine of the University of São Paulo, Travessa da R. Dr. Ovídio Pires de Campos 75, São Paulo, 05403-010 Brazil; 5 College of Life and Natural Sciences, University of Derby, Kedleston Road, Derby, KE22 1GB United Kingdom; 6 Wellcome Trust Centre for Neuroimaging, University College London, 12 Queen Square, London, WC1N 3BG United Kingdom

## Abstract

Understanding the brain mechanisms involved in diagnostic reasoning may contribute to the development of methods that reduce errors in medical practice. In this study we identified similar brain systems for diagnosing diseases, prescribing treatments, and naming animals and objects using written information as stimuli. Employing time resolved modeling of blood oxygen level dependent (BOLD) responses enabled time resolved (400 milliseconds epochs) analyses. With this approach it was possible to study neural processes during successive stages of decision making. Our results showed that highly diagnostic information, reducing uncertainty about the diagnosis, decreased monitoring activity in the frontoparietal attentional network and may contribute to premature diagnostic closure, an important cause of diagnostic errors. We observed an unexpected and remarkable switch of BOLD activity within a right lateralized set of brain regions related to awareness and auditory monitoring at the point of responding. We propose that this neurophysiological response is the neural substrate of awareness of one’s own (verbal) response. Our results highlight the intimate relation between attentional mechanisms, uncertainty, and decision making and may assist the advance of approaches to prevent premature diagnostic closure.

## Introduction

Understanding the brain mechanisms involved in the diagnosis of diseases – and prescription of medical treatments – may contribute to the development of methods that improve diagnostic accuracy and reduce errors in medical practice. We investigated the neural basis of diagnosis and prescription in two functional magnetic resonance imaging (fMRI) experiments with primary care physicians.

The first experiment was conducted to test the hypothesis that the brain systems involved in medical diagnosis are similar to those involved in identifying and naming things in everyday life. In the visual domain, we previously demonstrated that the diagnosis of radiological lesions engages neural systems very similar to those involved in naming animals^1^. Here, we test this hypothesis in the verbal domain, using textual sequences of diagnostic information. The comparison task was naming animals and objects. A second experiment tested the hypothesis that the prescription of medical treatments engages the same systems involved in the diagnosis of diseases.

Diagnosing diseases and prescribing treatments involve decision making under uncertainty^2^. Our third hypothesis was that activity in the frontoparietal attentional network (FPAN)^3–5^ is modulated by the diagnosticity (i.e., diagnostic salience) of available information, which reflects its ability to resolve uncertainty about the final diagnosis. For example, an unspecific symptom with low diagnosticity, e.g. fever, would evoke greater activity in the attentional network because there are many possible diagnoses that have to be entertained and excluded. Conversely, a positive HIV test – strongly associated with the diagnosis of AIDS – would engage this network to a lesser degree because the implicit diagnosis is relatively unique. The relation between symptom diagnosticity and brain activity was explored by manipulating the diagnostic specificity of information presented to participants.

In decision making, the sequential sampling of information leads to evidence accumulation until a confidence threshold is reached that triggers the decision process^6–8^. Evidence accumulation in a diagnostic investigation progressively decreases uncertainty regarding the final diagnosis. Our fourth hypothesis was that reduction of uncertainty, signaled by reduction of BOLD activity in the FPAN, constitutes an internal brain state that terminates evidence accumulation and thereby triggers a decision. In our experiments, decision making corresponds to vocalizing the diagnosis or treatment.

The most utilized methodology to investigate clinical reasoning has been clinical vignettes - short written descriptions of medical problems - followed by questions to be answered by participants^9^. In this experimental paradigm, each trial lasts for dozens of seconds; frequently for more than a minute^10–13^. The relatively long duration of the task trials using this paradigm creates special challenges for modelling the neural processes involved in diagnostic reasoning. To meet these demands, we employed a reductionistic approach, using an experimental design in which only key information needed to accomplish the tasks was conveyed by the stimuli.

**Figure 1:**
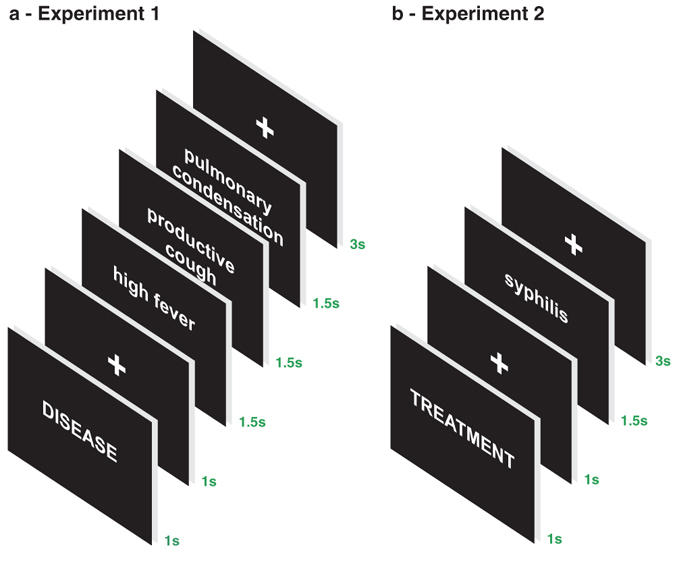
Temporal structure of the experiments. (**a**) Experiment 1: The temporal structure of a trial, totaling 9.5 s, comprised: 1- task signaling - diagnosing or naming - with the presentation of the words ‘DISEASE’, ‘ANIMAL’, or ‘OBJECT’ followed by a central cross in a black screen, totaling 2 s of foreperiod; 2- presentation of three pieces of information; 3- an additional period with a central cross in a black screen. (**b**) Experiment 2: The temporal structure the trial, totaling 6.5 s, was the following: 1- task signaling - disease diagnosis or treatment prescription - with the presentation of the words ‘DIAGNOSIS’ or ‘TREATMENT’ followed by a white central cross in a black screen, totaling 2 s of foreperiod; 2- presentation of the cue for the task, a diagnostic information or the name of a disease; 3- an additional period with a central white cross in a black screen. In this example, the expected response to syphilis is ‘penicillin’; its treatment.

In Experiment 1, written sequences with three pieces of medical diagnostic information were presented and participants were asked to name the disease that first occurred to them, without waiting for the end of the trial (Methods) (Fig. 1a). For example, ‘high fever’, ‘productive cough’, ‘pulmonary condensation’ for the target diagnosis of pneumonia. The comparison task was to name animals and objects based on a sequence of pertinent information, using the same format as the diagnostic task. For instance: ‘meow’ ‘domestic animal’ ‘black fur’ for cat as a target response. The stimuli followed a gradient of diagnosticity: from low to high diagnosticity or vice-versa (details in Methods). In Experiment 2, two types of stimuli were used: 1- diagnostic information to evoke the name of associated diseases; 2- names of diseases to elicit the associated treatments (Fig. 1b).

To estimate brain activity immediately preceding decisions, we increased the temporal resolution of the analyses with regressors that carefully modeled induced responses sampled by fMRI on a very fine timescale (details in Methods). This enabled the assessment of BOLD activity in epochs of 400 milliseconds (ms) over pre-response time (pre-RT) periods.

In brief, we demonstrate that diagnosing diseases, prescription of treatments, and naming animals/objects, using written information as stimuli, engage similar brain systems. Our results show that activity in the FPAN was modulated by the salience of the information presented, with low diagnosticity (high uncertainty) stimuli evoking greater responses. Highly diagnostic (low uncertainty) cues decreased activity in FPAN and may provide a correlate of premature diagnostic closure, an important cause of diagnostic errors. We observed a deactivation of the FPAN immediately preceding the final decision and vocalization of responses. This finding supports our hypothesis that reduction of uncertainty, signaled by activity in the FPAN, may participate in the selection of a final decision. Finally, we observed an unexpected and remarkable switch of BOLD responses, with greater activity within a right lateralized set of cortical areas and subcortical nuclei related to awareness in the 400 ms epoch at the onset of the response. We propose that this switch and concomitant BOLD responses in auditory monitoring regions are the neural correlates of becoming aware of one’s own responses.

## Results

### Behavioral results

Results for response times (RTs) and response durations in Exp. 1 are detailed in Table 1. There was no significant interaction in RTs between tasks and diagnosticity of the first stimulus [*F*(1,30) = 0.71, p = 0.41]. The mean RT for diagnosis of diseases, 3.76 s, was significantly greater as compared to naming animals and objects, 3.50 s [*F*(1,30) = 16.79, p < 0.001]. The mean RT for sequences in which the first stimulus had high diagnosticity, 3.12 s, was shorter relative to sequences with low diagnosticity, 4.15 s [*F*(1,30) = 352.67, p < 0.001]. Regarding the duration of the vocalization, there was no significant interaction related to first stimulus diagnosticity [*F*(1,30) = 0.37, p = 0.55]. The mean duration of the names of diagnoses vocalized, 0.80 s, was greater than the duration of the names of animals and objects, 0.59 s [*F*(1,30) = 324.24, p < 0.001]. The difference between the mean duration of responses when the first stimulus had high diagnosticity and when it had low diagnosticity - 0.70 s versus 0.69 s, respectively - was not significant [*F*(1,30) = 0.72, p = 0.40]. The low percentage of errors and sequences without responses, totaling 6.31% in the two tasks (Supplementary Table S1), indicates that participants had a near ceiling performance in this experiment.

**Table 1:** Mean response times and durations of the response vocalization in seconds*.

	Experiment 1				Experiment 2	
	Diagnosing		Naming		Diagnosing	Prescribing
	First stimulus diagnosticity					
	high	low	high	low		
Response time ( ± SD)	**3.23** (0.46)	**4.30** (0.38)	**3.00** (0.55)	**4.00** (0.25)	**1.98** (0.36)	**2.05** (0.32)
Duration ( ± SD)	**0.81** (0.13)	**0.80** (0.14)	**0.59** (0.09)	**0.59** (0.09)	**0.82** (0.12)	**0.92** (0.11)

*Errors, hesitations, more than one response, and outliers were excluded.

Abbreviation: SD, standard deviation.

In Exp. 2, one of the participants hesitated in 52.1% of the responses and his data were excluded from further analysis. The difference in the mean RT between both tasks was not statistically significant at p < 0.05 (*t* = 1.876, df 29, p = 0.071) (Table 1). However, the duration of responses in the diagnosis of diseases was significantly shorter than in the prescription of treatments (*t* = 11.96, df 29, p < 0.001) (Table 1). The percentage of errors and no responses was low in both tasks, diagnosis and prescription of treatments, totaling 9.17% (Supplementary Table S1), even though higher than in Exp. 1.

### Lexical semantic association results

In Exp. 1, the diagnosis of diseases evoked, on average, 3.93 different terms per target diagnosis. For example, in the sequence with cystitis as the target diagnosis, participants responded with ‘cystitis’, ‘urinary infection’, ‘UTI’, and ‘infection’. In sequences for naming animals and objects, there were on average 1.77 terms per target. The difference between both tasks in relation to the mean number of evoked words was statistically significant (*t* = 6.11, df 55, p < 0.001). While diagnosing diseases, participants gave more than one response in 4.1% of the events. This type of response with the vocalization of differential diagnosis was observed at least once in 25 (80.65%) participants.

As an example, a participant replied ‘hepatitis’ and ‘cirrhosis’ in a sequence with cirrhosis as the target response.

In Exp. 2, prescription of treatments elicited, on average, 6.48 terms per name of disease in contrast to 3.71 terms per diagnostic information in the diagnosis task, a statistically significant difference (*t = *5.50, df 55, p < 001). Eighteen (58.06%) participants verbalized differential diagnoses at least once in response to diagnostic information. For instance, in response to ‘despondency’, one participant answered ‘depression’ and ‘hypothyroidism’. In prescription of treatments, 17 (54.84%) participants responded with more than one treatment in at least one response. For example, in response to ‘giardiasis’ a participant replied with ‘albendazole’ and ‘metronidazole’, both correct treatments for this disease. Responses with task switch, e.g. verbalizing a treatment in reply to diagnostic information, occurred at least once for 18 (58.06%) participants. For example, two participants in response to the information ‘VDRL positive’, a diagnostic test for syphilis, replied ‘penicillin’, the treatment for syphilis.

### fMRI results

**Figure 2:**
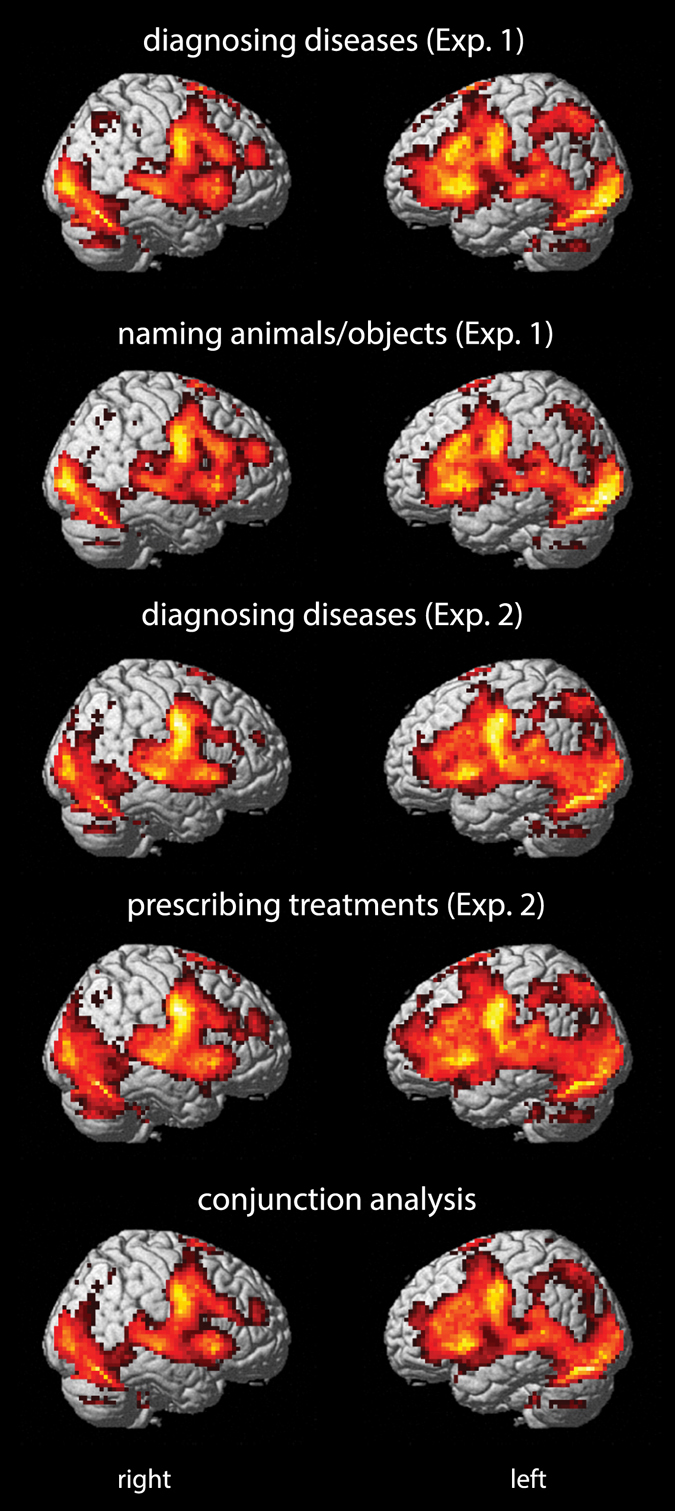
BOLD responses* to task effects in Experiments 1 and 2 versus control baseline and conjunction analyses of all tasks *p < 0.05 family-wise error (FWE) corrected; extent threshold k_E_ ≥ 10. Statistical parametric maps (SPMs) rendered on an International Consortium for Brain Mapping (ICBM) individual brain.

In Exp. 1, there were no significant interactions between tasks (diagnosing diseases and naming animals/objects) and first stimulus diagnosticity. We, therefore, report the main effects of task. In this experiment, there were three regions in the left hemisphere with greater BOLD responses in the contrast *diagnosing diseases* > *naming animals/objects*: a limited cluster (k_E_ = 127) encompassing posterior cingulate gyrus (peak level coordinates: −3, −43, 29; *t* = 7.75), precuneus (peak level coordinates: −6, −64, 32; *t* = 6.31), and a small area (k_E_ = 11) in angular gyrus (peak level coordinates: −36, −64, 56; *t* = 5.62). There was no suprathreshold activity in the reverse contrast, *naming animals/objects* > *diagnosing diseases*. In Exp. 2, there was only one small area (k_E_ = 19) in the left superior frontal gyrus (peak level coordinates: −12, 47, 51; *t* = 6.08) with suprathreshold activity in the contrast *prescribing treatments* > *diagnosing diseases*. No suprathreshold activity was detected in the reverse contrast, *diagnosing diseases* > *prescribing treatments*. Figure 2 shows the similarity between BOLD responses in the four tasks of the two experiments and the conjunction analysis with areas common to all tasks.

During the foreperiod in Exp. 1, when the type of task was signaled, the results of a one-way analysis of variance (ANOVA) *F* contrast between diagnosing diseases versus naming animals/objects showed no significant difference between both tasks. This suggests that our intervention to preclude differences in task set and performance anxiety was effective (see Methods).

**Figure 3:**
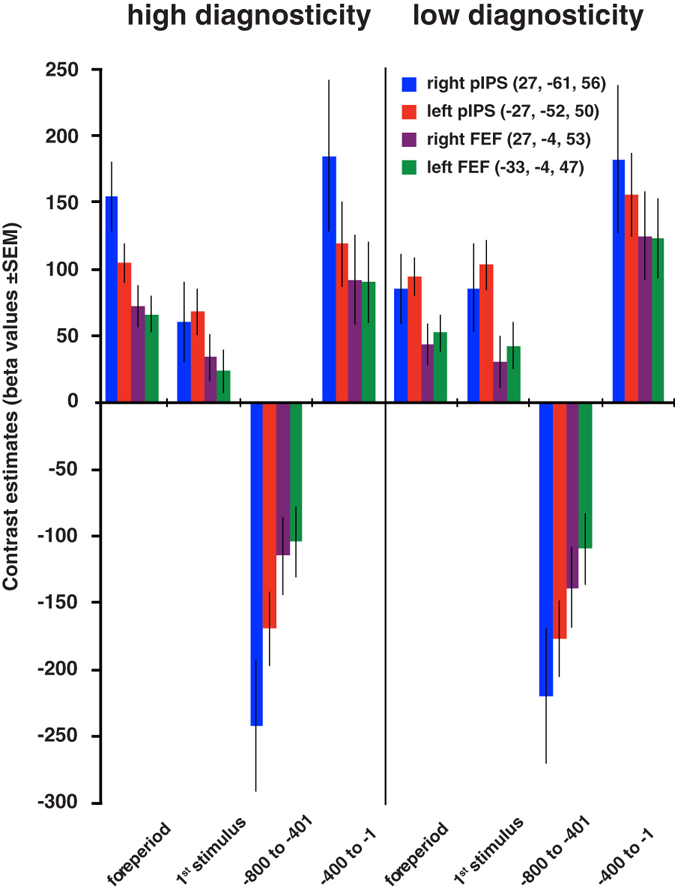
Experiment 1 contrast estimates in the frontoparietal attentional network in time periods* during tasks *400 ms epochs Abbreviations: FEF, frontal eye field; pIPS, posterior intraparietal sulcus; SEM, standard error of the mean. Foreperiod refers to task signaling in the beginning of the trial.

Results of an ANOVA of the initial 400 ms period of the foreperiod and the first stimulus revealed significant interactions between epoch and diagnosticity. There were no significant interactions between task and diagnosticity. Contrast estimates in Fig. 3 show that there was greater BOLD activity in the foreperiod epoch as compared to the high diagnosticity first stimulus epoch in all regions of the FPAN assessed. The differences between foreperiod and low diagnosticity first stimulus epochs were not significant. The same pattern of BOLD response was observed when tested under different tasks (Supplementary Fig. S1). In short, in functional attentional areas, highly informative diagnostic stimuli that reduce uncertainty caused reduction of BOLD response relative to the activity associated with the preceding attentional set. In the diagnosis task of Exp. 2, a comparison between BOLD activity in the foreperiod and presentation of information of high diagnosticity showed a similar pattern to the comparison between foreperiod and highly diagnostic first stimulus in Exp. 1 (Supplementary Fig. S2).

The epochs preceding behavioral responses were identified by accounting for the delay between cortical/electromyographic activity related to vocalization and the onset of the sound recording of the response, namely the RT (see Methods). This motivated our focus on the 400 ms epoch immediately preceding RT, from −400 to −1 ms, the beginning of vocalization. The preceding time period, from −800 to −401 ms, was considered to be an epoch late in the decisional period, prior to the decision implementation, the vocalization of responses. We consider that decisional processes began as soon as the first stimulus was processed.

In Exp. 1, the ANOVA comparing these two epochs, −800 to −401 ms versus −400 to −1 ms, revealed no significant interactions between diagnosticity and epoch, or diagnosticity and task. There was a limited interaction between epoch and task in the left intraparietal sulcus (peak level coordinates 30, −76, 32; k_E_ = 25) and right intraparietal sulcus (peak level coordinates 30, −79, 11; k_E_ = 12). These areas of interaction did not overlap with the intraparietal sulci areas of the FPAN detected in the present investigation.

In Exp. 1, differences between trials related to the duration/content of stimuli and intervening processes, e.g. working memory, could confound the results of the preRT epochs. To account for these possible confounds an additional analysis parameterising events with RTs was conducted; the results did not change.

In the decision period, from −800 to −401 ms, contrast estimates evidenced a sharp decrease in BOLD activity, with deactivation in the FPAN, preceding the beginning of the response in the epoch from −400 to −1 ms pre-RT (Fig. 3). The results showed the same pattern in both diagnosing and naming tasks (Supplementary Fig. S1) and in the tasks of Exp. 2 (Supplementary Fig. S2). The observed deactivation of this functional attentional network can therefore be plausibly attributed to confidence in subsequent decisions.

**Figure 4:**
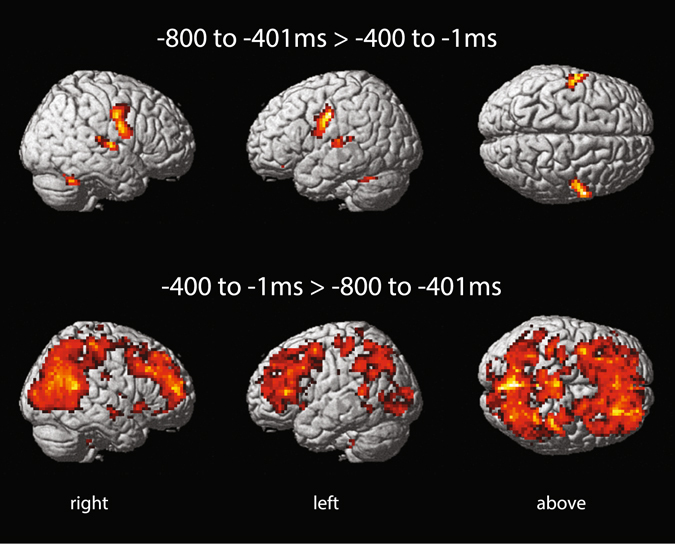
Experiment 1 BOLD effects* in contrasts of pre-RT epochs* p < 0.001 uncorrected for illustrative purposes; extent threshold k_E_ ≥ 10. SPMs rendered on an ICBM individual brain.

**Figure 5:**
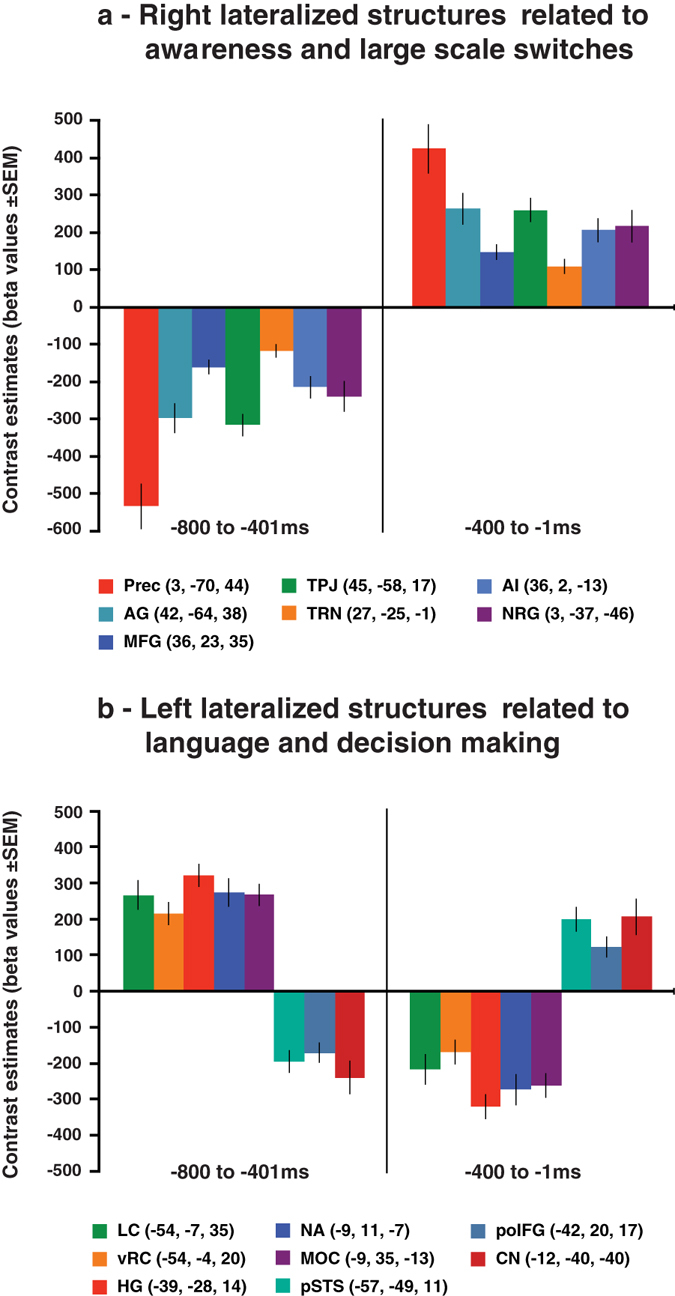
Experiment 1 contrast estimates in pre-RT epochs. Abbreviations: (**a**) **AG**, angular gyrus; **AI**, anterior insula; **MFG**, middle frontal gyrus; **NRG**, nucleus reticularis gigantocellularis; **Prec**, precuneus; **TPJ**, temporoparietal junction; **TRN**, thalamic reticular nucleus; (**b) CN**, cochlear nucleus; **HG**, Heschl’s gyrus; **LC**, laryngeal cortex; **MOC**, medial orbital cortex; **NA**, nucleus accumbens; **poIFG**, pars opercularis inferior frontal gyrus; **pSTS**, posterior superior temporal sulcus; **vRC**, ventral Rolandic cortex.

The comparison of the decision epoch versus the response epoch showed a dramatic change of BOLD activity (Fig. 4). In the contrast −*800 to* −*401* *ms* > −*400 to* −*1* *ms*, greater BOLD activity was restricted to bilateral posterior superior temporal gyrus including Heschl’s gyrus, bilateral areas in the precentral gyrus, right cerebellar hemisphere lobule VI, left medial orbital cortex, and left nucleus accumbens/ventral striatum. In the contrast −*400 to* −*1* *ms* > −*800 to* −*401* *ms*, there was greater BOLD activity in a large cortical system, involving mainly lateral and medial frontoparietal areas. In Exp. 2, a similar pattern of activity was observed in these contrasts (Supplementary Fig. S3). Contrast estimates in Fig. 5 detail the change of BOLD activity between these epochs in a right lateralized set of cortical areas and subcortical nuclei involved in awareness and large scale switches of brain activity^14–19^, left lateralized structures involved in language^20, 21^, and decision making^22^ in addition to the FPAN areas described above. The same pattern held when contrasts estimates were discriminated by tasks in Exp. 1 (Supplementary Fig. S4) and Exp. 2 (Supplementary Fig. S5).

## Discussion

The striking similarity between BOLD responses in Experiments 1 and 2 and the commonalities disclosed by the conjunction analysis (Fig. 2) support our hypothesis that diagnosing diseases, prescribing medical treatments, and naming objects/animals based on written information are subserved by similar neural systems. The same pattern of contrast estimates in 19 different brain areas, during four time epochs (Supplementary Figs S1, S2, S4 and S5) further corroborate our initial hypotheses. The results are also in agreement with our previous study in the visual domain, with radiological diagnosis^1^.

Several fMRI studies have investigated diagnostic reasoning using clinical vignettes and/or multiple-choice questions^11–13^. However, the approaches adopted in those studies preclude the identification of brain networks involved in the diagnostic process per se. Downar *et al*.^23^ investigated the learning process in prescribing treatments with a dual choice associative learning paradigm. They focused on the comparison between high versus low performers. Conversely, in the current study, we studied prescription of treatments that participants already knew (i.e., that they had already learned). Furthermore, our tasks were not planned to compare between different levels of expertise.

We proposed that the cognitive mechanism through which diagnostic information (symptoms, clinical signals, laboratory data, etc.) evoke diagnoses is a lexical semantic associative process^1^. The semantic representation of diagnostic information, i.e. their meaning, is associated with the lexical representation of diseases, i.e. their names. Salient diagnostic information not only evokes the names of diseases associated with it but also related information, e.g. treatments. That is, there is an automatic and unconscious activation of the semantic network of concepts associated with the diagnostic information presented^24^. Responses with two alternative differential diagnoses in both experiments, or responding with treatments in reply to diagnostic cues in Exp. 2 indicate that information associated with diseases (symptoms, differential diagnoses, treatments, etc.) is organized in lexical semantic associative networks that are activated during the diagnostic process. It is of note that differential diagnoses were evoked in a time frame of few seconds. A similar finding was observed in our study on radiological diagnosis^1^. The implicit organization of semantic networks and lexical semantic associative processes have been studied with different models^25, 26^.

The foreperiod, when participants were informed of the impending task, can be conceptualized as the moment when the doctor is about to assess a patient without any prior information. It constitutes the period of greatest uncertainty in both experiments. Low diagnosticity information, e.g. fever, may evoke more diagnostic alternatives^27^, thereby increasing uncertainty about the final diagnosis. When compared to foreperiod epochs, highly diagnostic information was associated with a reduction of BOLD activity in the FPAN (Fig. 3, and Supplementary Figs S1 and S2). That is, greater uncertainty is associated with greater activity in the FPAN.

The relation between BOLD activity in the FPAN and attentional demands in lexical semantic tasks has been investigated in fMRI studies^28, 29^. Electrophysiological studies have also addressed the relationship between attention and lexical semantic processing^30, 31^. Furthermore, modulation of attentional brain mechanisms by uncertainty has been studied in perceptual tasks within a predictive coding framework^32^. Interestingly, in the context of active inference, an increase in the precision or certainty over response options is a key component in action selection. A growing literature suggests that this may be mediated by neuromodulatory mechanisms (e.g. dopaminergic projections) that induce a winner-takes-all like selection of competing responses^33, 34^. This selection process may be reflected here in terms of reduced synaptic activity –and haemodynamic responses– in regions representing the consequences of competing decisions, e.g. cortical motor areas involved in the articulation of responses, in a way that may not be dissimilar to biased competition^35^ and representational sharpening^36^.

In clinical practice, the reduction of uncertainty by informative cues is an important aspect of efficient diagnosis, avoiding protracted and unnecessary diagnostic investigations. However, ironically, there are situations in which physicians find highly diagnostic information at the beginning of the assessment of a patient, make a diagnosis, and conclude the diagnostic investigation prematurely; without detecting other important pathological conditions. As an example, a patient with tiredness, despondency, and low thyroxin level receives a correct diagnosis of hypothyroidism but her/his depressive disorder is not diagnosed. Premature closure is a common cause of diagnostic errors^37^. Errors related to satisfaction of search (SOS) in radiology are considered to be similar to premature closure^38^. One of the mechanisms proposed to explain SOS errors is that ‘obvious abnormalities capture visual attention and decrease vigilance for more subtle abnormalities’ ^39^.

The implicit attentional modulation by uncertainty may be the underlying neural substrate of this premature cessation of evidence accumulation: Highly diagnostic information may decrease FPAN monitoring and may precipitate premature diagnosis. Uncertainty also depends on the physician’s knowledge and clinical context. For example, a VDRL positive test is strongly associated with the diagnosis of syphilis; however, false-positive results of this test occur in a variety of other conditions (e.g. several bacterial and viral infections, connective tissue diseases, old age, etc.). Depending on the physician’s knowledge about this test and the clinical context, s/he may entertain or not the possibility of a false-positive VDRL test and the differential diagnoses associated with it. One approach to prevent premature closure is to counterbalance the reduction of uncertainty related to highly diagnostic information at the beginning of the assessment. For example, Kostopolou *et al*.^40^ demonstrated that the presentation of a list of differential diagnoses with a computerized diagnostic support system in the beginning –as compared to the end of the diagnostic assessment– improved diagnostic accuracy of primary care physicians. Augmenting uncertainty with a list of alternative diagnoses increases implicit attention and may help prevent premature closure.

Our behavioral results indicate that decision making was related to a reduction of uncertainty regarding diagnosis as evidence accumulated. RTs in sequences beginning with highly diagnostic information in Exp. 1 were 1.25 s longer in comparison to diagnosis of diseases in Exp. 2, also based on highly diagnostic information (Table 1). This suggests that participants in Exp. 1 awaited subsequent information to confirm the diagnosis before responding, despite being instructed to respond as soon as the diagnosis occurred to them. In sequences beginning with highly diagnostic information, RTs were 1.03 s shorter as compared to sequences with low diagnosticity of the first stimulus. This suggests that decrease of uncertainty associated with high diagnosticity information, as compared to less salient information, speeded decision making in Exp. 1 (Table 1).

In Experiments 1 and 2, contrast estimates in brain structures involved in decision making - medial orbital cortex and nucleus accumbens - showed greater BOLD activity from −800 to −401 ms and a subsequent deactivation from −400 to −1 ms pre-RT (Fig. 5a, and Supplementary Figs S4 and S5). The reverse pattern was observed in a right lateralized set of frontoparietal cortical areas, including the FPAN, and subcortical nuclei involved in awareness and large scale switches of brain activity^14–19^ (Figs 3 and 5a, and Supplementary Figs S4 and S5). A large scale switch was also observed in the contrast estimates of areas involved in language (Fig. 5b, and Supplementary Figs S4 and S5). These findings are compatible with a switch between a decisional mode, during the epoch from −800 to −401 ms, to a vocalization mode in the epoch from −400 to −1 ms pre-RT.

Deactivation of the FPAN in the epoch in the end of the decisional period – in relation to the vocalization epoch – in both experiments, supports our hypothesis that reduction of uncertainty signaled by this network may participate in the trigger of decision making (Fig. 3, and Supplementary Figs S1 and S2). Our conjecture is that the FPAN deactivation would directly or indirectly disinhibit motor areas mediating the vocalization of the response^41^. In addition, we propose that areas involved in language and lexical semantic processing modulate activity in the FPAN. These hypotheses can be further investigated using dynamic causal modeling^42^.

The patterns of BOLD activity in the vocalization epoch, in areas involved in auditory feedback, indicate how responses are self-monitored (Fig. 5b, and Supplementary Figs S4 and S5). Deactivation of Heschl’s gyrus during this epoch is compatible with the observed attenuation of responses within the auditory cortex to self-produced speech relative to listening^43^. This reflects the generic phenomena of sensory attenuation; i.e. attenuation of responses to the sensory consequences of self-made acts^44^. Greater BOLD activity in Wernicke’s area, which encompasses the posterior superior temporal sulcus, is in agreement with its involvement in monitoring speech production^45, 46^. Increased BOLD response in the cochlear nuclei in this epoch is compatible with an exteroceptive monitoring of self-produced speech. The pattern of BOLD activity in these three areas before vocalizations were audible suggests they received predictive information (i.e., corollary discharge) as decisions were made^43, 44, 46^.

An unexpected and remarkable finding was the large scale switch of BOLD activity in a set of right lateralized cortical areas and subcortical nuclei involved in awareness in the vocalization epoch as compared to the preceding decisional epoch (Figs 4 and 5a, and Supplementary Figs S4 and S5). The frontoparietal cortical areas that showed a shift from deactivation to activation in the pre-RT periods have been implicated in awareness in multiple cognitive domains^14, 17^. The same applies to the engagement of anterior insula^18, 47^. The thalamic reticular nucleus, which has a functional topographic organization, is involved in the modulation of thalamocortical circuits and large scale switches of brain activity^19^; its peak level coordinates in our study (Fig. 5, and Supplementary Figs S4 and S5) are at the same coronal plane of the medial geniculate body, the auditory thalamus. Activity in nucleus reticularis gigantocellularis, situated in the reticular formation at the pontomedullary junction in the brain stem, has been related to arousal and cortical activation in multimodal tasks^16^. Attention and awareness are distinct but interacting processes with attention being considered a possible gateway to awareness^14, 17, 48^. In agreement with this view, BOLD activity in the FPAN areas and awareness related structures followed the same pattern, deactivation from −800 to −401 ms followed by activation from −400 to −1 ms (Figs 3 and 5a, and Supplementary Figs S1, S2, S4, and S5). The correlations between attention and awareness during decision making in the verbal domain need now to be investigated with experimental designs that establish their putative causal relations.

Activity in auditory monitoring areas and greater BOLD activity in right lateralized structures related to awareness and attention in the vocalization epoch lead us to think that these networks are the neural substrates which mediate awareness of self generated responses. It has been proposed that we need to hear, aloud or silently, our speech to become aware of our verbal thoughts^49–52^. Overt speech and inner speech, notwithstanding implementation differences, engage similar neural networks^21, 46^. In a behavioral study, auditory feedback was considered necessary to infer the meaning of the vocalized responses^52^. In a magnetoencephalography (MEG) study of the intention to speak, participants reported the onset of speech 54 ms before the beginning of the recorded utterance, the RT^53^. To report the start of the speech, participants needed to be aware of their responses^17^. The delay between cortical/electromyographic records and RT varied between 200 and 400 ms (see Methods). That is, participants in the MEG study possibly became aware of their responses after vocalization had already initiated. Both studies, despite methodological limitations, provide indirect support for our hypothesis.

The characterization of brain mechanisms engaged in cases of straightforward diagnosis may lead to the development of experimental protocols to study the diagnosis of clinical cases of greater complexity that calls on greater diagnostic expertise. One important element that is missing in our experiments is the active investigative process that occurs during a diagnostic assessment. Our experimental design was not planned to directly address questions related to the existing theoretical models of diagnostic reasoning in the domain of cognitive psychology^54, 55^. Instead, we focused on hypotheses not explicitly included in these models.

From a broader perspective, our results contribute to the understanding of the intimate relationship between attentional or salience mechanisms, uncertainty, and the processes involved in decision making. The conjecture regarding the presumed neural substrate of response awareness may contribute to the investigation of how people become aware of their own verbal responses. Finally, our time resolved modeling of the BOLD response opens new possibilities in experimental protocols using fMRI.

## Methods

### Participants

Neuroimaging data were collected from 35 physicians working in primary care; four had their data excluded for distinct reasons (details and recruitment criteria in Supplementary Information). The remaining 31 participants, 21 male, had an average age of 38.6 years (SD ± 10.0) (range 28–64) with an average of 13.9 years (SD ± 9.8) (range 3–39) of medical practice. The participation was not rewarded monetarily. The research protocol was approved by the ethics committees of the Faculty of Medicine of the University of São Paulo and the Albert Einstein Israelite Hospital and followed the institutional ethics guidelines. Participants signed an informed consent.

### Tasks

Creation and testing of medical stimuli were supervised by medical doctors with expertise in internal medicine and family medicine. Stimuli were selected based on their capacity to evoke predefined responses. The low error rates (Supplementary Table S1) support the validity of the stimuli within the context of the tasks.

The tasks of the two experiments were assessed in four stages of pilot tests with physicians that did not participate in the final experiments: an initial stage, testing the stimuli with written responses; two stages to optimize the content and the temporal structure of the tasks, with the presentation of the stimuli in the screen of a notebook computer; and a final stage with tests in the MRI scanner. Overall, 23 physicians participated in those tests; 19 were second and third year residents in internal medicine.

The order of the experiments was counterbalanced between participants. The total duration of the fMRI data collection – with an event related design – was 38 min 45 s divided into four sessions.

### Experiment 1

The first stimulus on each trial was a piece of information with high or low diagnosticity. The diagnosticity of the last stimulus was the reverse of the first one. The middle stimulus could have either low or high diagnosticity. The narrative structure of the information was restricted to enable the balancing of lexical variables between the different types of stimuli sequences and minimize syntactic variables that could confound the results (Supplementary Table S2). To minimize the influence of medical diagnostic expertise, a potentially confounding variable, medical sequences were created to produce ceiling performance. In the creation of diagnostic stimuli, qualitative measures were used instead of numerical values, e.g. ‘high fever’, to avoid the confounding variable related to the cognitive processing of numbers.

The final selection, after the pilot tests, encompassing 56 sequences for diseases, 28 for animals, and 28 for objects is detailed in Supplementary Information. Eight sequences for diseases, 4 sequences for animals, and 4 sequences for objects were used for training. Seven sets of stimuli were created to enable the rotation of stimuli used in training and minimize order effects of stimuli presentation (details in Supplementary Information).

Forty-eight null events with a white central cross in a black screen for 9.5 s were introduced to create a low level control baseline. The 48 events for diagnosis of diseases, 24 events for naming animals, 24 events for naming objects, and 48 null events totaled 1,368 s, divided into two sessions of equal duration.

Participants were asked to vocalize their decisions as soon as they occurred to them; without waiting for the end of the presentation of the sequence. They were oriented to give their responses as succinctly as possible, including the use of acronyms. They were also instructed to correct the verbal response if they changed their minds during the course of the event.

### Experiment 2

After pilot tests, 56 diagnostic cues strongly associated with the diagnosis of a disease and 56 names of diseases were selected (details in Supplementary Information). Eight stimuli from each type were used in training. Seven sets of stimuli were created to enable the rotation of stimuli used in training as in Exp. 1. Lexical balancing between both types of stimuli was conducted as in Exp. 1 (Supplementary Table S2). There were 48 null events with a white central cross in a black screen for 6.5 s. The 48 events for diagnosis, 48 events for treatment prescription, and 48 null events comprised 936 s, divided into two sessions of equal duration. Training was realized immediately before the data collection (details in Supplementary Information).

### Assessment and management of performance anxiety

In the pilot tests, we observed that medical tasks evoked performance anxiety in some subjects. For this reason, participants’ anxiety levels were monitored during the fMRI sessions using an assessment scale. When increased anxiety was detected, a brief abdominal breathing exercise was used to control it (details in Supplementary Information).

### Data collection

Presentation of the stimuli and recording of responses was carried out using E-Prime 2.0 software (Psychology Software Tools Inc.). An optical fiber FOMRI-III microphone (Optoacoustics Ltd.) was used to register the responses.

Magnetic resonance images were collected in a Siemens Trio 3 tesla system (Siemens AG) with a 12 channel head coil. The head of the participants was immobilized using a built-in vacuum cushion. BOLD sensitive T2* functional images were obtained using prospective motion correction (PACE) gradient-echo echoplanar pulse sequence employed to minimize effects of head movements with the following parameters: time of repetition (TR): 2.31 s, time of echo (TE): 30 ms, flip angle: 90°, field of view (FOV): 206 × 206 mm, and in plane resolution: 3 × 3 mm. Forty-three axial slices with 3 mm in width with an inter slice gap of 0.6 mm were acquired in ascending order, parallel to the inter-commissural plane. B_0_ images were obtained in the interval between the second and third sessions. After the experimental sessions, a T1 structural image was acquired using a magnetization-prepared rapid acquisition gradient-echo (MPRAGE) sequence with TR: 2.5 s, TE: 3.45 ms, flip angle: 7°, FOV: 256 × 256 mm, with isotropic voxels of 1 mm^3^.

### Data analyses

Measurement of RTs and duration of response vocalization were performed using the acoustic waveform of the responses with Audacity 2.04 software (http://audacityteam.org) after filtering the background noise.

Assessment of the behavioral responses was conducted separately by two medical doctors following a standardized protocol. A response was considered correct if it was compatible with the information presented. In case of discordance, the reviewers reached a consensus agreement. Only the researcher in charge of the data collection knew the identity of participants.

The behavioral results of Exp. 1 were submitted to a full factorial 2 × 2 ANOVA: task type (diagnosing and naming) and diagnosticity of the first stimulus (high and low). In Exp. 2 the behavioral results of the two tasks were analyzed with a paired *t* test.

Processing and statistical analyses of the functional images were conducted using SPM12 software (Wellcome Trust Centre for Neuroimaging)^56^. Functional images were corrected for static distortions using field maps created with B_0_ images and changes in those distortions caused by head motion during the realignment step. Structural images were processed using a unified segmentation procedure implementing tissue segmentation, bias correction, and spatial normalization, and coregistered to functional images. Deformations fields calculated during the segmentation procedure were applied to normalize functional images to the Montreal Neurological Institute (MNI) space in 3 mm^3^ voxels. Smoothing was carried out with an isotropic Gaussian kernel with 6 mm full width at half maximum (FWHM).

Head movements, assessed during the realignment of functional images during preprocessing, were generally small with intra-session translational and rotational movements < 1 mm and < 1°, respectively.

After preprocessing, two types of analyses were conducted at the within-subject level to estimate stimulus-specific BOLD responses at each voxel. First, analyses encompassing the whole duration of the decision making process to compare responses between 1- diagnosing diseases versus naming animals/objects, 2- diagnosing diseases versus prescribing treatments. Second, to test our hypotheses regarding diagnosticity of stimuli and brain activity preceding the decision, we analyzed time segments within events (see below).

In both analyses, time series from each voxel were high-pass filtered with a cut-off period of 1/128 Hertz to remove signal drift and low-frequency noise. Six-parameter spatial transformations of the realignment of functional images were introduced in general linear models (GLMs) as regressors of no interest to account for head movements. Temporal correlations were modeled with a first order autoregressive model with white noise. A gray matter image resulting from the segmentation of the structural image was used as a mask in the analyses of BOLD activity. Events with errors, absence of response, hesitations, more than one response, outliers (>1.5 box plot length), and events in which there was superposition of the previous event response were excluded from the analyses – by being modeled as events of no interest.

### Time series models

In analyses involving the whole trial, events were modeled with boxcar functions and convolved with a BOLD canonical hemodynamic response function. In Exp. 1 GLMs were created for each task, diagnosis or naming, on each level of the first stimulus, high and low diagnosticity. In Exp. 2 there were two GLMs, one for each task: diagnosis of diseases and prescription of treatments.

The correlation between RT and duration of vocalization was calculated. Their Spearman correlation coefficients in Exps. 1 and 2 were 0.05 and 0.08, respectively. Due to the lack of significant correlation, both variables were modeled as parametric effects in all GLMs. The resulting subject-specific contrast images were taken to standard second level (random effects) between-subject analyses.

We expected a difference among stimulus types in both experiments in terms of lexical associations between cues for diagnosis, naming, and treatments. This means there could be a difference in the mean number of possible responses for the cues in both experiments. The lexical semantic results confirmed that supposition. These differences and their impact on lexical semantic processing influence BOLD responses^57^. We therefore developed a lexical ambiguity index to account for between subject differences. We used two metrics to calculate this index: 1- the sum of responses with hesitations, no response, more than one response, and outliers for each task type per participant as an indirect estimate of ambiguity for each subject; 2- the mean number of different words produced in the responses for each type of event in each task, as a proxy to the lexical association network related to each cue. The lexical ambiguity index calculated for each task type and each subject corresponded to the product of these metrics. In both experiments, the lexical ambiguity index was used as a covariate in the second level analyses.

To identify systematic responses during decision making that were conserved over subjects, an analysis of covariance (ANCOVA) of contrasts from Exp. 1 was conducted with a full 2 × 2 factorial design: 1- type of task: diagnosis and naming; 2- first stimulus diagnosticity: low and high. BOLD contrasts in Exp. 2 were assessed with a one-way ANCOVA with task type as a factor with two levels, diagnosis of diseases and prescription of treatments. A conjunction analysis was used to identify common areas of BOLD activation in the four tasks of the two experiments based on the minimum *t* statistic^58^.

### Modeling of responses at first (within-subject) level

To increase the temporal resolution, enabling analyses of time segments within events, we employed regressors that used the temporal structure of volume acquisition: 43 slices and ascending order. We defined epochs during the pre-RT period and, to do this, it was necessary to account for the delay between brain activity related to the articulation of the response and the subsequent sound detection, the RT. Studies using direct measurements of electrocorticographic activity during vocalization suggest a delay of approximately 200–300 ms between neuronal responses in ventral Rolandic cortex and perceptual sound detection^45, 59^. Furthermore, the results of a study using electromyography (EMG) in a verbal Stroop task showed a delay of approximately 350–400 ms between the start of the EMG activity – time-locked to RT – and the response time per se^60^. On the basis of these findings, we performed pairwise contrasts of BOLD activity between epochs with durations ranging from 200 to 500 ms. A 400 ms epoch was identified as the most sensitive (i.e., statistically efficient) duration to model BOLD activity in relation to RT. Two epochs were demarcated: a period from −400 to −1 ms preceding RT, corresponding to the onset of vocalization, and an epoch from −800 to −401 ms preceding RT, which was considered a time epoch within the decision period, encompassing the end of the decisional process. In Exp. 1, a 2 × 2 × 2 full factorial ANOVA was specified: 1- epoch (decision versus vocalization), 2- task type (diagnosis versus naming), and 3- diagnosticity of the first stimulus (high versus low). In Exp. 2, a 2 × 2 full factorial ANOVA was conducted: 1- epoch (decision versus vocalization) and 2- task type (diagnosis versus prescription). Epochs were modeled as boxcar functions.

Using short epochs during specific periods of peristimulus time is possible because the event related model of fMRI responses exploits the fine sampling of peristimulus time, when averaging event related responses. This follows because the TR does not have a fixed relationship with the underlying neuronal and haemodynamic responses preceding RT. This means peristimulus time is sampled in a hyperacute fashion. In effect, a difference between subsequent (decisional and response) epochs corresponds to a switch (increase or decrease) in neuronal activity at the point when evidence accumulation gives way to response implementation. The ability to model these responses over several hundred milliseconds is based upon the same fMRI modeling that has been previously shown to detect shifts in the latency of neuronal responses in the order of 200 ms^61^. In support of the validity of the temporal resolution our modeling approach, a recent study demonstrated that the onset of vascular responses to neural activity can be very fast; in the order of hundreds of milliseconds^62^.

The analyses of the pre-RT epochs were applied to functional images reprocessed with 2 mm^3^ voxels and smoothing with a 4 mm FWHM kernel to better identify subcortical structures. Specialized neuroanatomy atlases were employed to localize the ensuing effects^63–65^.

The remaining comparison was between brain activity in the foreperiod in which participants were informed of the task ahead (e.g., diagnosing or naming), relative to the presentation of the first stimulus, with low or high diagnosticity in Exp. 1. Areas of the FPAN were defined in the contrast foreperiod > first stimulus. The peak level coordinates of the frontal eye fields, near the junction of the superior frontal sulcus with the precentral gyrus, are compatible with the localization of these areas in other fMRI studies^66, 67^. The initial 400 ms within the foreperiod and the first stimulus were modeled as boxcar functions. Responses to the first stimulus were also modeled parametrically in terms of number of the syllables in the initial stimulus. In Exp. 1, a 2 × 2 × 2 full factorial ANOVA was conducted: time epoch (foreperiod versus first stimulus), task type (diagnosis versus naming), and diagnosticity of the first stimulus (high versus low). In Exp. 2, a 2 × 2 full factorial ANOVA was conducted: 1- epoch (decision versus vocalization) and 2- task type (diagnosis versus prescription).

BOLD activity results are presented with a statistical criterion of p < 0.05 family-wise error (FWE) corrected using random field theory, unless otherwise stated. Peak level coordinates are in the MNI space. Response estimates were based on the parameters of GLMs (beta values ± standard error of the mean, SEM) using an *F* contrast at the peak coordinates in the brain structures of interest. The same peak level coordinates used in Exp.1 were employed in response estimates in Exp. 2; with exception of the thalamic reticular nucleus (Exp. 1: 27, −25, −1 versus Exp. 2: 24, −31, 5) because it was not possible to find a significant contrast in Exp. 2 using Exp. 1 coordinates.

Finally, we conducted a one-way ANOVA during the 2 s epoch of the foreperiod in Exp. 1 to assess possible effects of performance anxiety when diagnosing diseases versus naming animals/objects.

## Electronic supplementary material

Supplementary Information
