# Supplementary material for: How doctors diagnose diseases and prescribe treatments: an fMRI study of diagnostic salience
Source: Sci Rep. 2017 May 2;7:1304. doi: 10.1038/s41598-017-01482-0 (PMC5430984; doi:10.1038/s41598-017-01482-0)
Supplement: Supplementary file 1 — Supplementary Information [file 41598_2017_1482_MOESM1_ESM.pdf]

Supplementary Information

How doctors diagnose diseases and prescribe treatments: an fMRI study of diagnostic salience

Authors: Marcio Melo[1,2]*, Gustavo D.F. Gusso[3], Marcelo Levites[3], Edson Amaro Jr.[2,4], Eduardo Massad[1,5], Paulo A. Lotufo[3], Peter Zeidman[6], Cathy J. Price[6], Karl J. Friston[6]

1- Laboratory of Medical Investigations, LIM-01, Faculty of Medicine of the University of São Paulo, São Paulo, Brazil;
2- Albert Einstein Israelite Hospital, IIEP, São Paulo, Brazil;
3- Department of Internal Medicine, Faculty of Medicine of the University of São Paulo, São Paulo, Brazil;
4- Department of Radiology, Faculty of Medicine of the University of São Paulo, São Paulo, Brazil;
5- College of Life and Natural Sciences, University of Derby, Derby, United Kingdom;
6- Wellcome Trust Centre for Neuroimaging, University College London, London, United Kingdom.

* Corresponding author. E-mail: marciomelo@me.com

1- Supplementary Methods
2- Supplementary Figures S1-S5
3- Supplementary Tables S1-S2
4- Stimuli for Experiment 1
5- Stimuli for Experiment 2

1- Methods

Participants

The recruitment criteria of participants were: 1- working part time or full time in primary care - general internal medicine or family medicine - and completed medical residence; 2- having Portuguese as first language; 3- right handedness, assessed with a modified version of the Edinburgh Handedness Inventory ; 4- absence of neurological and/or psychiatric disorders in the present and not in use of psychopharmacologic or neurological medication. Most physicians were affiliated with the medical facilities of the Faculty of Medicine of the University of São Paulo and the Albert Einstein Israelite Hospital.

Four participants had their data excluded for the following reasons: 1- intense headache during the data collection; 2- distortions in fMRI images provoked by dental amalgam; 3- complaint of intense jitteriness during data collection; 4- intense somnolence with several micro naps during data collection.

Experiment 1

Altogether, 79 sequences with the diagnosis of different diseases as a target, 36 sequences for naming different animals, and 40 sequences for naming different objects were assessed in the pilot tests. The final selection is detailed in item 4.

The organization of the sets obeyed the following criteria:

1- Order of the sequences, including sequences in the training, was pseudorandomized in such a way to avoid more than two events of the same type occurring in succession;

2- The presence of a high or low diagnosticity stimuli in the beginning of the sequence was counterbalanced so that half of the sequences began with one of the two types of stimuli;

3- The order of the sequences in relation to the diagnosticity of the first stimulus was also pseudorandomized avoiding more than three sequences with the same type of first stimulus;

4- Two versions were created for each of the seven sets of sequences, with the inversion of the initial and the end stimuli; one set beginning with a low diagnosticity and the other with a high diagnosticity stimulus, totaling 14 sets.

Experiment 2

Two lists of stimuli were created and assessed in the pilot tests: 1- 112 diagnostic information selected from the sequences used in Exp.1; 2- 78 names of diseases. The final selection is detailed in item 5.

The order of the stimuli was pseudorandomized; with no more than three tasks of the same type in succession.

Training

The training order of the two experiments followed the order of the experiments in the scanner. Participants received written instructions for the tasks. They were trained to minimize movements while vocalizing the responses.

The training sequences of 16 events and eight null events for each experiment were presented twice to the participants in a notebook computer using E-Prime 2.0. In the first run, the presentation pace was controlled by the

researcher for an initial familarization. In the second run, the pace followed the temporal structure of the experiment.

Assessment and management of performance anxiety

We used an assessment scale presented verbally in which participants were asked to rate their grade of tension: zero represented 'complete relaxation' and 10 'high tension'. The use of the term anxiety in this assessment was purposefully avoided. The assessment was carried out during the training, before the beginning of the data collection inside the scanner and in the intervals between fMRI sessions.

After reporting tension greater than 3, 18 participants (58,2%) were oriented to do abdominal breathing. For 11 (35.5%), the relaxation was conducted before data collection. For seven (22.7%), it was necessary to realize the relaxation between, at most, two functional sessions. All 18 participants presented reduction of tension to ≤3 after brief periods of relaxation exercises supervised by the researcher in charge of the data collection.

Data collection

The stimuli in size 50 Arial font were retro-projected using a Sanyo ProtraX Multiverse projector (Sanyo Electric Co.) onto a translucent screen and viewed through a mirror in the head coil. The resulting visual angles ranged from 6.0º to 26.0º in width and from 1.4º to 4.9º in height, depending on the number and extend of the words in each stimulus.

3- Supplementary Figures

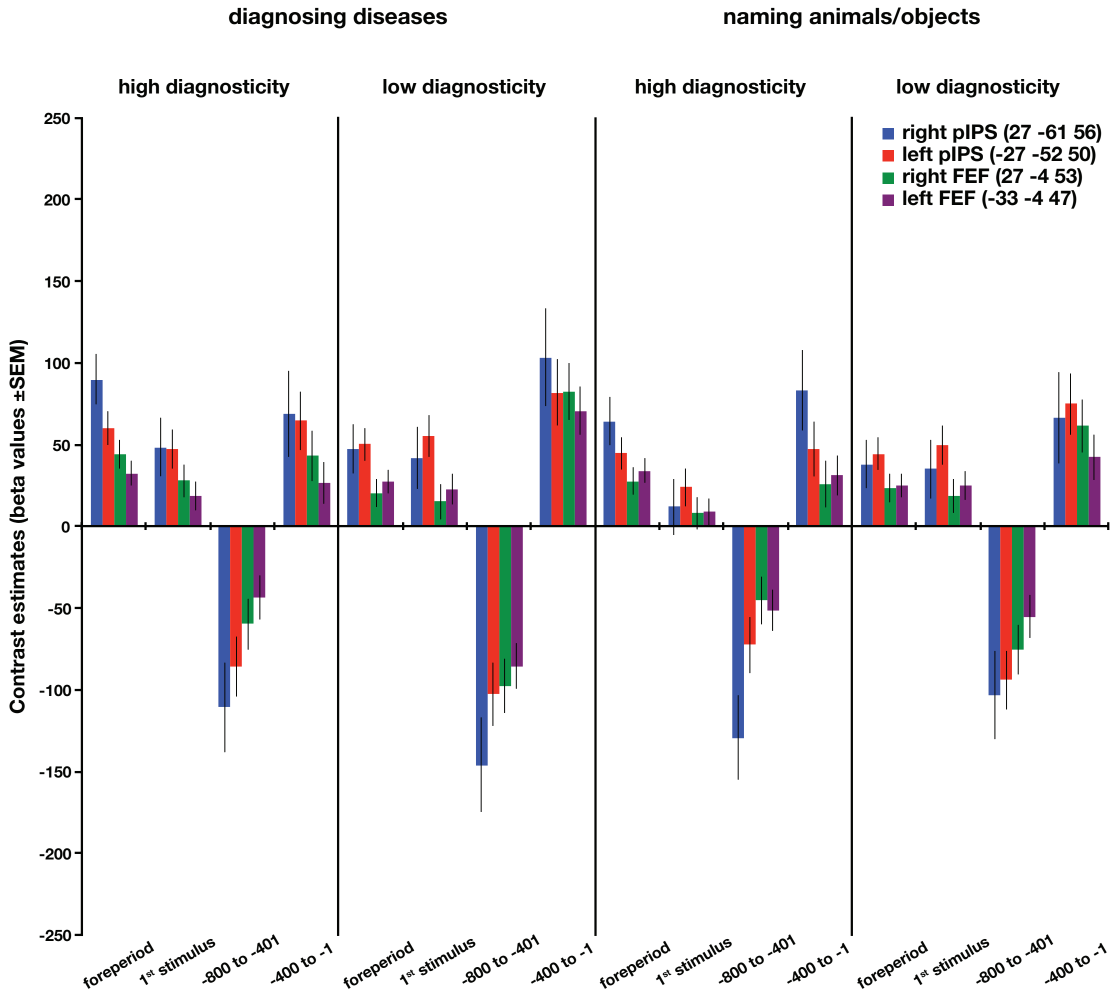

Supplementary Figure S1- Experiment 1 contrast estimates in the frontoparietal attentional network during time periods* discriminated by tasks

* 400 ms epochs

Abbreviations: FEF, frontal eye field; pIPS, posterior intraparietal sulcus. Foreperiod refers to task signaling in the beginning of the trial

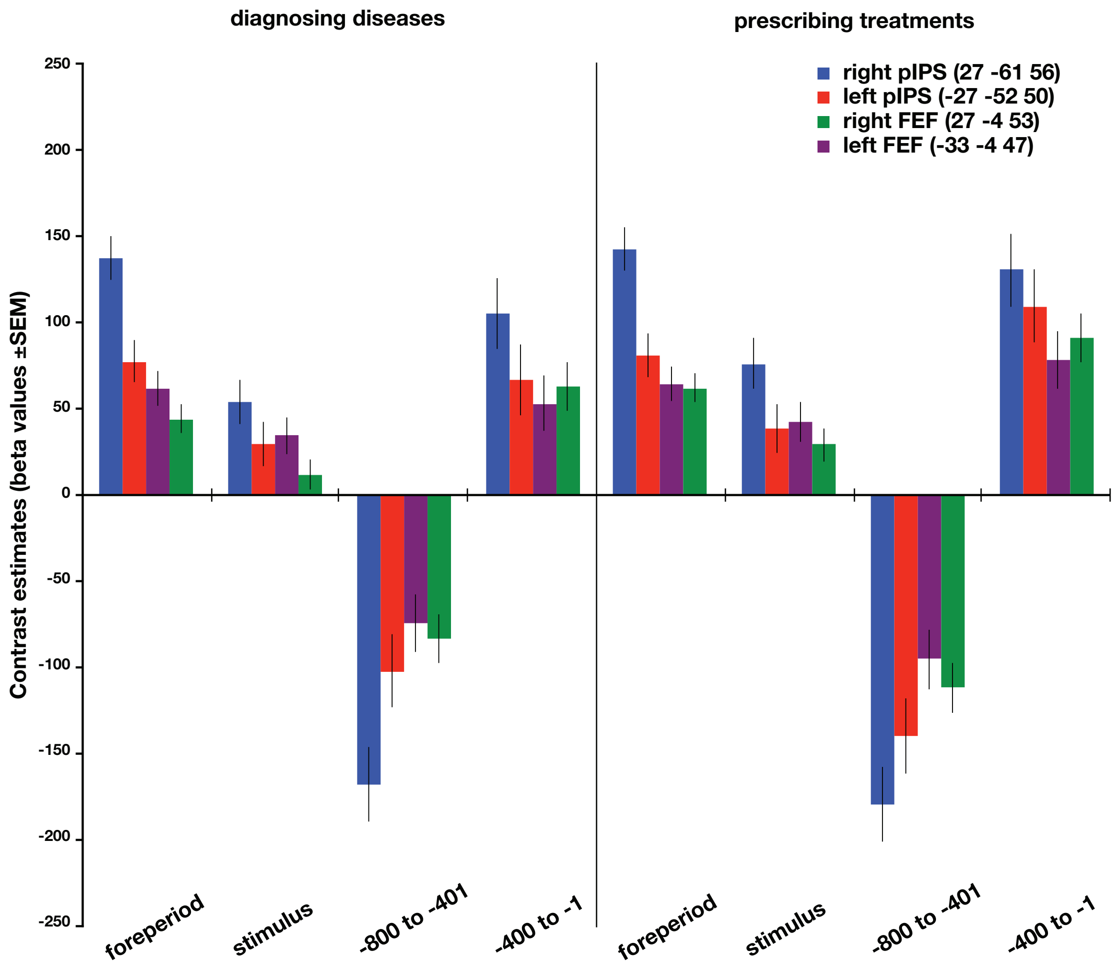

Supplementary Figure S2- Experiment 2 contrast estimates in the frontoparietal attentional network during time periods* discriminated by tasks

*400 ms epochs

Abbreviations: FEF, frontal eye field; pIPS, posterior intraparietal sulcus. Foreperiod refers to task signaling in the beginning of the trial

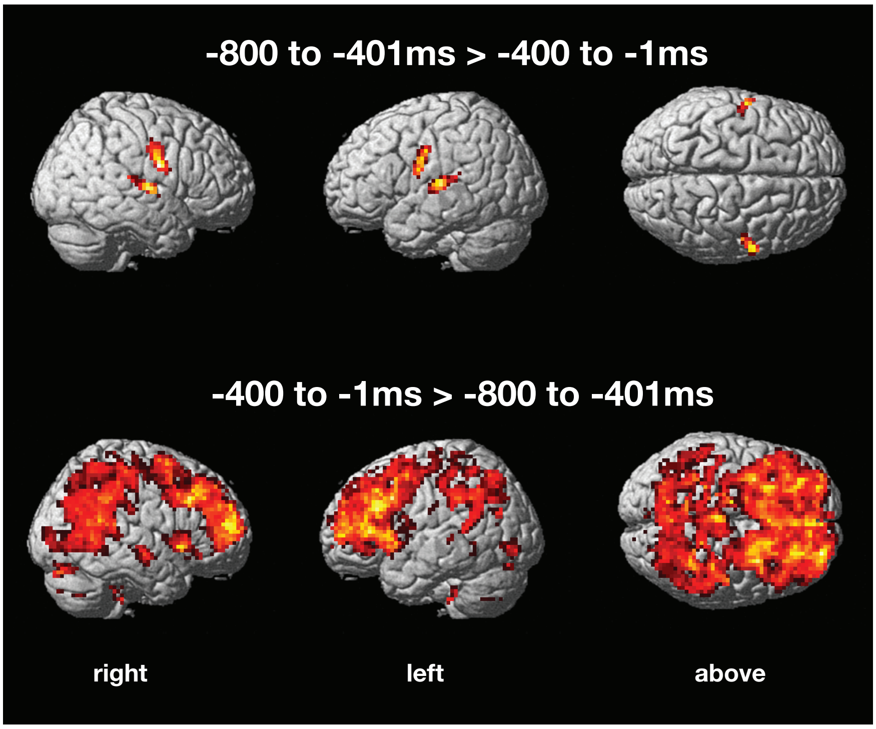

Supplementary Figure S3- Experiment 2 BOLD effects* in contrasts of pre-RT epochs

*$p<0.001$ uncorrected for illustrative purposes; extent threshold $k_E \geq 10$. SPMs rendered on an ICBM individual brain.

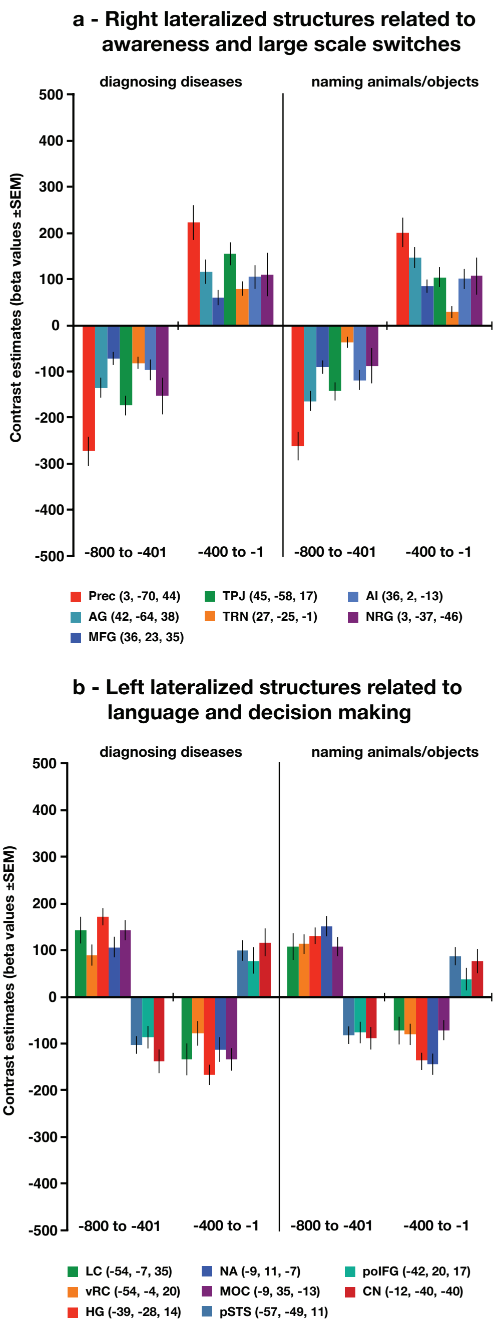

Supplementary Figure S4- Experiment 1 contrast estimates in pre-RT epochs discriminated by tasks.

Abbreviations: **a- AG**, angular gyrus; **AI**, anterior insula; **MFG**, middle frontal gyrus; **NRG**, nucleus reticularis gigantocellularis; **Prec**, precuneus; **TPJ**, temporoparietal junction; **TRN**, thalamic reticular nucleus; **b- CN**, cochlear nucleus; **HG**, Heschl's gyrus; **LC**, laryngeal cortex; **MOC**, medial orbital cortex; **NA**, nucleus accumbens; **poIFG**, pars opercularis inferior frontal gyrus; **pSTS**, posterior superior temporal sulcus; **vRC**, ventral Rolandic cortex .

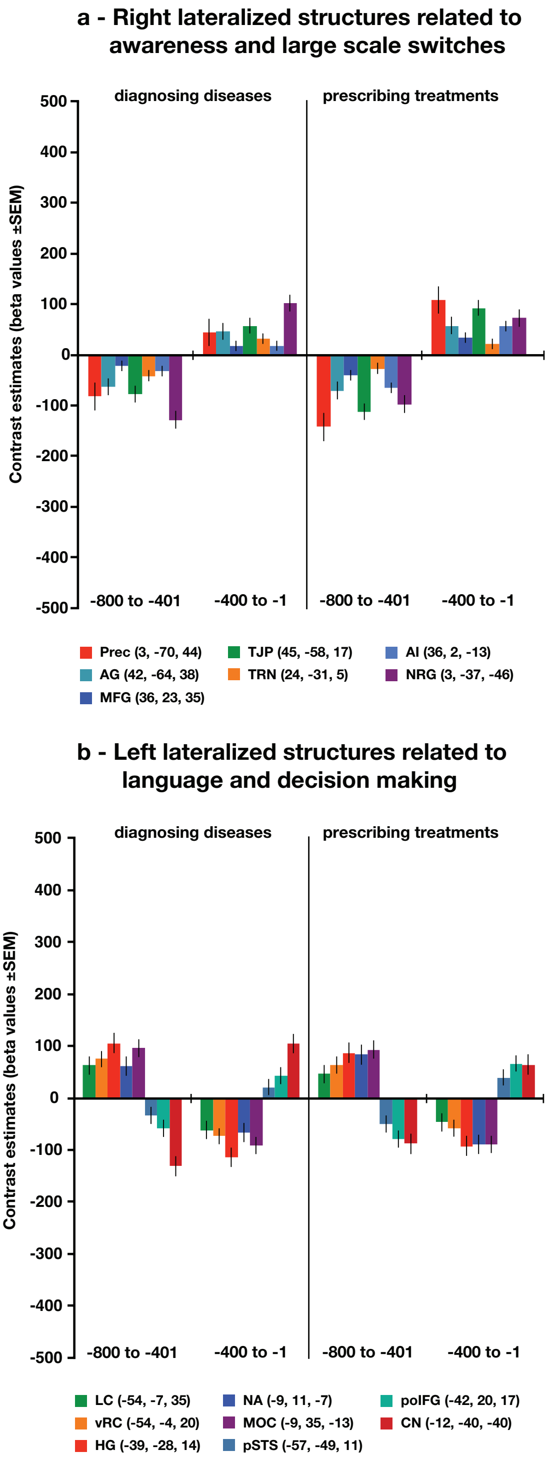

Supplementary Figure S5- Experiment 2 contrast estimates in pre-RT epochs discriminated by tasks.

Abbreviations: **a- AG**, angular gyrus; **AI**, anterior insula; **MFG**, middle frontal gyrus; **NRG**, nucleus reticularis gigantocellularis; **Prec**, precuneus; **TPJ**, temporoparietal junction; **TRN**, thalamic reticular nucleus; **b- CN**, cochlear nucleus; **HG**, Heschl's gyrus; **LC**, laryngeal cortex; **MOC**, medial orbital cortex; **NA**, nucleus accumbens; **poIFG**, pars opercularis inferior frontal gyrus; **pSTS**, posterior superior temporal sulcus; **vRC**, ventral Rolandic cortex .

3- Supplementary Tables

	Experiment 1			Experiment 2		
**Type of response**	**Diagnosing**	**Naming**	**ANOVAs* F(1, 30) (p)**	**Diagnosing**	**Prescribing**	**Paired *t* test (df=55) (p)**
**Errors**	**2.15**	**1.61**	**0.27**	**2.22**	**2.92**	**0.41**
**No response**	**0.87**	**1.68**	**0.02**	**1.11**	**2.92**	**0.01**
**Hesitations**	**3.97**	**2.42**	**0.05**	**3.96**	**5.97**	**0.01**
**Two or more responses**	**4.10**	**1.57**	**0.13**	**3.75**	**6.11**	**0.01**
**Outliers**	**1.41**	**0.98**	**0.82**	**2.29**	**2.85**	**0.19**
**Task switch**	**-**	**-**	**-**	**3.82**	**0.76**	**<0.01**

Supplementary Table S1- Types of responses (in percentage)*

* There were no significant interactions between tasks, diagnosis and naming, and diagnosticity of the first stimulus in each of the response types discriminated. For this reason the results are reported for the main effect of task type.

	Experiment 1*		Experiment 2*	
	**Diseases sequences**	**Animals and objects sequences**	**Diagnostic stimuli**	**Names of diseases**
**Words**	**6.66** (1.73)	**6.71** (1.16)	**2.14** (0.90)	**1.88** (0.81)
**Syllables**	**18.64** (4.01)	**17.77** (3.45)	**6.66** (2.11)	**6.38** (2.60)
**Nouns**	**3.52** (1.01)	**3.38** (0.78)	**1.23** (0.43)	**1.16** (0.37)
**Adjectives**	**1.50** (0.91)	**1.50** (1.01)	**0.64** (0.62)	**0.57** (0.63)
**Verbs**	**0.57** (0.66)	**0.70** (0.71)	-	-
**Words of other grammatical classes** #	**1.07** (1.01)	**1.14** (0.92)	**0.27** (0.45)	**0.16** (0.37)

Supplementary Table S2- Lexical balancing

* mean number per sequence (±SD)
# words of other grammatical classes with predominately syntactic functions: prepositions, contractions, articles, conjunctions, pronouns, and numerals.

4- Set of stimuli for Experiment 1

To help the visualization of the task, stimuli are placed in the order in which they were presented to participants using one of the sets employed in the experimental protocol, including training stimuli. In the first column of the table is the temporal order of the presentation of sequences in seconds. Null events are marked with crosses. High diagnosticity stimuli in the beginning or in the end of the sequence are in bold.

Training

Temporal sequence	1st information	2nd information	3rd information	*target diagnosis or name*
0	animal ovíparo<br>oviparous animal	coberta de penas<br>covered with feathers	**cacarejos**<br>**clucks**	***galinha***<br>***hen***
9.5	+	+	+	
19	**transaminases elevadas**<br>**elevated transaminases**	icterícia<br>jaundice	queixa-se de náusea<br>complains of nausea	***hepatite***<br>***hepatitis***
28.5	mulher de meia-idade<br>middle-aged woman	irregularidades menstruais<br>menstrual irregularities	**fogachos**<br>**hot flashes**	***menopausa***<br>***menopause***
38	+	+	+	
47.5	inseto<br>insect	produz mel<br>produces honey	**construção de colmeias**<br>**honeycomb building**	***abelha***<br>***bee***
57.0	**regurgitação**<br>regurgitation	queimação retroesternal<br>retrosternal burning	irritação na garganta<br>throat irritation	***refluxo gastro-esofágico***<br>***gastroesophageal reflux***
66.5	+	+	+	
76	cabo plástico<br>plastic handle	prevenção de cáries<br>caries prevention	**limpeza dos dentes**<br>**teeth cleaning**	***escova de dente***<br>***toothbrush***
85.5	+	+	+	
95	preservação de alimentos<br>food preservation	alimentos perecíveis<br>perishable food	**refrigeração**<br>**refrigeration**	***geladeira***<br>***refrigerator***
104.5	+	+	+	
114	**corrimento esbranquiçado**<br>**whitish vaginal discharge**	prurido vaginal<br>vaginal pruritus	hiperemia vaginal<br>vaginal hyperemia	***candidíase***<br>***candidiasis***
123.5	pulseira metálica<br>metalllic band	formato redondo<br>round format	**marca horas**<br>**time indicator**	***relógio de pulso***<br>***wristwatch***
133	**tromba comprida**<br>**long trunk**	orelhas grandes<br>large ears	animal vegetariano<br>vegetarian animal	***elefante***<br>***elephant***
142.5	poliúria<br>polyuria	polidipsia<br>polydypsia	**hiperglicemia**<br>**hyperglycemia**	***diabetes***<br>***diabetes***
152	+	+	+	
161.5	**TSH diminuído**<br>**low TSH**	tem sudorese<br>has sudoresis	sente palpitação<br>complains of palpitation	***hipertireoidismo***<br>***hyperthyroidism***
171	+	+	+	
180.5	**produz veneno**<br>**produces venom**	chocalho na cauda<br>rattle in the tail	animal rastejante<br>crawling animal	***cascavel***<br>***rattlesnake***
190	+	+	+	
199.5	linfonodo axilar<br>axillary lymph node	retração de mamilo<br>nipple retraction	**nódulo mamário**<br>**breast nodule**	***câncer de mama***<br>***breast cancer***
209	colocado no pescoço<br>worn around the neck	enfeite pessoal<br>personal ornament	de pedras preciosas<br>of precious stones	***colar***<br>***necklace***
218.5	**dor precordial recorrente**<br>**recurring precordial pain**	exercício desencadeia dor<br>exercise provokes pain	colesterol elevado<br>elevated cholesterol	***angina pectoris***<br>***angina pectoris***

First session

Temporal sequence	1st information	2nd information	3rd information	*target diagnosis or name*
0	tosse prolongada<br>prolonged cough	caverna pulmonar<br>pulmonary cavern	**PPD positivo**<br>**positive PPD**	***tuberculose***<br>***tuberculosis***
9.5	+	+	+	
19	**varrição**<br>**sweeping**	cabo comprido<br>long handle	limpeza doméstica<br>housecleaning	***vassoura***<br>***broom***
28.5	+	+	+	
38	**urina de rato**<br>**rat urine**	icterícia<br>jaundice	tem febre<br>has fever	***leptospirose***<br>***leptospirosis***
47.5	tem febre<br>has fever	cefaleia<br>headache	**rigidez de nuca**<br>**nuchal rigidity**	***meningite***<br>***meningitis***
57	+	+	+	
66.5	**hemoglobina baixa**<br>**low hemoglobin**	palidez de mucosas<br>pale mucosa	dispneia aos esforços<br>dyspnea on exertion	***anemia***<br>***anemia***
76	telhado<br>roof	paredes de tijolo<br>brick walls	**para morar**<br>**to reside**	***casa***<br>***house***
85.5	+	+	+	
95	hemoptise<br>hemoptysis	dispneia súbita<br>sudden dyspnea	**d-dímero aumentado**<br>**elevated d-dimer**	***embolia pulmonar***<br>***pulmonary embolism***
104.5	+	+	+	
114	**come formigas**<br>**eats ants**	focinho comprido<br>long snout	animal peludo<br>furry animal	***tamanduá***<br>***anteater***
123.5	de couro<br>of leather	colocada no bolso<br>put in pockets	**carrega dinheiro**<br>**holds money**	***carteira***<br>***wallet***
133	**queimação epigástrica**<br>**epigastric burning**	dor melhora com comida<br>pain gets better with food	melena<br>melena	***úlcera péptica***<br>***peptic ulcer***
142.5	+	+	+	
152	tem febre alta<br>has high fever	dificuldade para engolir<br>difficulty swallowing	**dor de garganta**<br>**throat pain**	***amigdalite***<br>***amygdalitis***
161.5	corpo alongado<br>elongated body	veneno potente<br>potent venom	**ferrão na cauda**<br>**sting in the tail**	***escorpião***<br>***scorpion***
171	+	+	+	
180.5	**coaxa**<br>**croaks**	animal anfíbio<br>amphibious animal	animal saltador<br>hopping animal	***sapo***<br>***frog***
190	**disúria**<br>**dysuria**	urgência urinária<br>urinary urgency	dor suprapúbica<br>suprapubic pain	***cistite***<br>***cystitis***
199.5	+	+	+	
209	**hiperemia conjuntival**<br>**conjunctival hyperemia**	prurido nos olhos<br>ocular pruritus	fotofobia<br>photophobia	***conjuntivite***<br>***conjunctivitis***
218.5	+	+	+	
228	marcha arrastada<br>dragging gait	hipomimia facial<br>hypomimia	**tremor nas mãos**<br>**shaking hands**	***doença de Parkinson***<br>***Parkinson's disease***
237.5	+	+	+	
247	**para escrever**<br>**to write**	tinta azul<br>blue ink	papel em branco<br>white paper	***caneta***<br>***pen***
256.5	redução de reflexos<br>reflexes reduction	dor em queimação<br>burning pain	**parestesia em bota**<br>**stocking paresthesia**	***neuropatia periférica***<br>***peripheral neuropathy***
266	+	+	+	

275.5	poliartrite<br>polyarthritis	eritema em borboleta<br>butterfly rash	**FAN positivo**<br>**positive ANF**	***lupus eritematoso sistêmico***<br>***systemic lupus erythematosus***
285	**medo súbito**<br>**sudden fear**	queixa-se de palpitação<br>complains of palpitation	tem dispneia<br>has dyspnea	***síndrome do pânico***<br>***panic disorder***
294.5	de couro<br>of leather	amarra com cadarço<br>fastens with shoelaces	**para andar**<br>**to walk**	***sapato***<br>***shoes***
304	+	+	+	
313.5	**coberto por escamas**<br>**covered with scales**	respiração por guelras<br>breathe through gills	animal aquático<br>aquatic animal	***peixe***<br>***fish***
323	animal quadrúpede<br>quadruped animal	animal roedor<br>rodent animal	**coberto de espinhos**<br>**covered with spines**	***porco-espinho***<br>***porcupine***
332.5	+	+	+	
342	**novelas**<br>**soup operas**	controle remoto<br>remote control	aparelho eletrônico<br>electronic equipment	***televisão***<br>***television***
351.5	+	+	+	
361	animal mamífero<br>mammiferous animal	voa à noite<br>flies at night	**moradia em cavernas**<br>**lives in caverns**	***morcego***<br>***bat***
370.5	**vive em esgotos**<br>**lives in sewers**	transmissor de doenças<br>carrier of diseases	animal roedor<br>rodent animal	***rato***<br>***mouse***
380	+	+	+	
389.5	tem náusea<br>complains of náusea	cólica no hipocôndrio direito<br>right upper quadrant pain	**fosfatase alcalina elevada**<br>**elevated alkaline phosphatase**	***colelitíase***<br>***cholelithiasis***
399	alça para transporte<br>handle to carry	para viagem<br>to travel	**guardar roupas**<br>**transports clothes**	***mala***<br>***suitcase***
408.5	+	+	+	
418	**queixa-se de vertigem**<br>**complains of vertigo**	zumbido<br>tinnitus	tem náusea<br>complains of nausea	***labirintite***<br>***labirintitis***
427.5	tem náusea<br>complains of nausea	amenorreia<br>amenorrhea	**beta HCG elevada**<br>**increased beta HCG**	***gravidez***<br>***pregnancy***
437	**bolsa para filhote**<br>**purse for cub**	animal saltador<br>hopping animal	pelo marrom<br>brown coat	***canguru***<br>***kangaroo***
446.5	+	+	+	
456	**perda de memória progressiva**<br>**progressive memory loss**	idoso<br>elderly	tem apatia<br>has apathy	***demência***<br>***dementia***
465.5	tem febre<br>has fever	dores no corpo<br>body aches	**congestão nasal**<br>**nasal congestion**	***gripe***<br>***influenza***
475	+	+	+	
484.5	**controle de temperatura**<br>**temperature control**	quente frio<br>hot cool	acionamento eletrônico<br>electronic operation	***ar-condicionado***<br>***air-conditioner***
494	+	+	+	
503.5	pelos longos<br>long fur	animal doméstico<br>domestic animal	**latido estridente**<br>**strident bark**	***cachorro***<br>***dog***
513	tem tosse<br>has cough	tem dispneia<br>has dyspnea	**chiados**<br>**wheezes**	***asma***<br>***asthma***
522.5	**troponina aumentada**<br>**increased troponinin**	dor precordial<br>precordial pain	tem sudorese<br>has sudoresis	***infarto de miocárdio***<br>***myocardial infarction***
532	+	+	+	
541.5	pelos negros<br>black fur	animal doméstico<br>domestic animal	**miado**<br>**meow**	***gato***<br>***cat***
551	**alergia a pólen**<br>**pollen allergy**	obstrução nasal<br>nasal obstruction	rinorréia clara<br>clear rhinorhea	***rinite***<br>***rhinitis***
560.5	pele seca<br>dry skin	mostra-se cansado<br>appears tired	**TSH aumentado**<br>**increased TSH**	***hipotireoidismo***<br>***hypothyroidism***
570	+	+	+	

579.5	**alavanca de câmbio** **gear shift**	volante redondo round steering wheel	pneus de borracha rubber tyres	***carro*** ***car***
589	**ideação suicida** **suicidal thoughts**	desanimado despondent	mostra-se cansado appears tired	***transtorno depressivo*** ***depressive disorder***
598.5	+	+	+	
608	cabo plástico plastic handle	parafuso metálico metallic screw	**aparafusar** **to screw**	***chave de fenda*** ***screwdriver***
617.5	**cozinha alimentos** **cooks food**	botijão de gás gas cyilinder	acendedor automático automatic lighter	***fogão*** ***stove***
627	animal quadrúpede quadruped animal	animal vegetariano vegetarian animal	**listras pretas e brancas** **black and white stripes**	***zebra*** ***zebra***
636.5	+	+	+	
646	**tentáculos longos** **long tentacles**	ventosas múltiplas multiple suckers	animal marinho marine animal	***polvo*** ***octopus***
655.5	cabo de madeira wood handle	cabeça de aço metal head	**bate pregos** **strikes nails**	***martelo*** ***hammer***
665	**comportamento sexual de risco** **risky sexual behaviour**	candidíase de repetição repetitive candidiasis	pneumonia de repetição repetitive pneumonia	***SIDA*** ***AIDS***
674.5	+	+	+	

Second session

**Temporal sequence**	**1st stimulus**	**2nd stimulus**	**3rd stimulus**	***target diagnosis or name***
0	tem febre has fever	rinorreia purulenta purulent rhinorrhea	**dor no rosto** **facial pain**	***sinusite*** ***sinusitis***
9.5	+	+	+	
19	**tomar banho** **to take bath**	funcionamento elétrico electrical operation	higiene pessoal personal hygiene	***chuveiro*** ***shower***
28.5	**emagreceu muito** **great weight loss**	sangramento intestinal intestinal bleeding	obstipação obstipation	***câncer de cólon*** ***colon cancer***
38	+	+	+	
47.5	**tabagismo** **tabagism**	tosse crônica chronic cough	tem dispneia has dyspnea	***DPOC*** ***COPD***
57	cabo de madeira wood handle	lâmina de metal metal blade	**afiada** **sharp**	***faca*** ***knife***
66.5	+	+	+	
76	possui casco has carapace	retrai a cabeça retracts the head	**caminha devagar** **moves slowly**	***tartaruga*** ***turtle***
85.5	**enxugar o corpo** **to dry the body**	tecido absorvente absorbent tissue	tecido felpudo fluffly tissue	***toalha*** ***towel***
95	alcoolismo alcoholism	dor abdominal intensa intense abdominal pain	**amilase aumentada** **increased amylase**	***pancreatite*** ***pancreatitis***
104.5	+	+	+	
114	**moradia em chiqueiros** **lives in sties**	carne comestível edible meat	animal doméstico domestic animal	***porco*** ***pig***
123.5	funcionamento eletrônico electronic operation	aparelho portátil portable equipment	**comunicação interpessoal** **interpersonal communication**	***telefone celular*** ***cellphone***
133	+	+	+	

142.5	**ascite**<br>**ascites**	varizes esofágicas<br>esophageal varices	icterícia<br>jaundice	***cirrose***<br>***cirrhosis***
152	**vesículas no tórax**<br>**vesicles in the thorax**	lesões em dermátomo<br>dermatomal lesions	dor em queimação<br>burning pain	***herpes zoster***<br>***herpes zoster***
161.5	+	+	+	
171	animal mamífero<br>mammiferous animal	pelos macios<br>soft fur	**gosta de cenoura**<br>**likes carrots**	***coelho***<br>***rabbit***
180.5	+	+	+	
190	tem febre alta<br>has high fever	tosse produtiva<br>productive cough	**condensação pulmonar**<br>**pulmonary condensation**	***pneumonia***<br>***pneumonia***
199.5	tem febre<br>has fever	otorréia<br>otorrhea	**dor de ouvido**<br>**earache**	***otite média***<br>***otitis media***
209	+	+	+	
218.5	**cefaléia latejante**<br>**throbbing headache**	aura visual<br>visual aura	tem náusea<br>complains of nausea	***enxaqueca***<br>***migraine***
228	**produz lã**<br>**produces wool**	balidos<br>bleats	animal quadrúpede<br>quadruped animal	***ovelha***<br>***sheep***
237.5	+	+	+	
247	hipertensão arterial<br>arterial hypertension	síncope<br>syncope	**hemiparesia súbita**<br>**sudden hemiparesis**	***AVC***<br>***stroke***
256.5	+	+	+	
266	dor de cabeça<br>headache	tem febre baixa<br>has low fever	**aumento de parótidas**<br>**increased parotids**	***caxumba***<br>***mumps***
275.5	**usada para sentar**<br>**used to seat**	encosto elevado<br>elevated back	de madeira<br>of board	***cadeira***<br>***chair***
285	+	+	+	
294.5	**gosta de banana**<br>**likes banana**	pendura-se nas árvores<br>hangs in trees	animal mamífero<br>mammiferous animal	***macaco***<br>***monkey***
304	**fator reumatoide**<br>**rheumatoid fator**	rigidez pela manhã<br>morning stiffness	dores articulares<br>joint pains	***artrite reumatoide***<br>***rheumatoid arthritis***
313.5	+	+	+	
323	hemoptise<br>hemoptysis	fumante<br>smoker	**emagreceu muito**<br>**great weight loss**	***câncer de pulmão***<br>***lung cancer***
332.5	tampa<br>lid	de alumínio<br>of aluminium	**cozinha alimentos**<br>**cooks food**	***panela***<br>***pan***
342	+	+	+	
351.5	**creatinina aumentada**<br>**elevated creatinine**	anúria<br>anuria	edema palpebral<br>palpebral edema	***insuficiência renal***<br>***renal insufficiency***
361	**pedra na urina**<br>**stone in urine**	hematúria<br>hematuria	dor abdominal intensa<br>intense abdominal pain	***nefrolitiase***<br>***nephrolithiasis***
370.5	+	+	+	
380	anemia<br>anemia	esplenomegalia<br>splenomegaly	**febre terçã**<br>**tertian fever**	***malária***<br>***malaria***
389.5	+	+	+	
399	fica suspenso<br>stays hanged	secagem de roupas<br>to dry clothes	**pendurar roupas**<br>**to hang clothes**	***varal***<br>***clothes line***
408.5	**água parada**<br>**stagnant water**	tem febre alta<br>has high fever	dores no corpo<br>aching body	***dengue***<br>***dengue***
418	+	+	+	
427.5	**voa alto**<br>**flies high**	piloto<br>pilot	meio de transporte<br>transport medium	***avião***<br>***airplane***
437	prurido no pé<br>foot pruritus	sapato fechado<br>closed shoes	**frieiras**<br>**'frieiras' is a popular Brazilian term for interdigital skin cracking in the feet**	***dermatomicose***<br>***dermatomycosis***
446.5	+	+	+	
456	**bicho barbeiro**<br>**barber bug (Brazilian popular term for Chagas disease's insect vector)**	cardiomegalia<br>cardiomegaly	queixa-se de cansaço<br>complains of tiredness	***doença de Chagas***<br>***Chagas disease***

465.5	animal quadrúpede<br>quadruped animal	animal vegetariano<br>vegetarian animal	**pescoço comprido**<br>**long neck**	***girafa***<br>***giraffe***
475	+	+	+	
484.5	cabo plástico<br>plastic handle	armação metálica<br>metallic scaffold	**proteção contra chuva**<br>**rain protection**	***guarda-chuva***<br>***umbrella***
494	**relinchos**<br>**neighs**	crina longa<br>long mane	animal quadrúpede<br>quadruped animal	***cavalo***<br>***horse***
503.5	exantema<br>rash	cancro duro<br>hard chancre	**VDRL positivo**<br>**positive VDRL**	***sífilis***<br>***syphilis***
513	+	+	+	
522.5	**proteção contra sol**<br>**protection against the sun**	usado na cabeça<br>used in the head	de palha<br>of straw	***chapéu de palha***<br>***straw hat***
532	**dupla corcova**<br>**double hump**	habitante do deserto<br>desert inhabitant	pelo marrom<br>brown coat	***camelo***<br>***camel***
541.5	+	+	+	
551	voa alto<br>flies high	totalmente preto<br>completely dark	**come carniça**<br>**eats carrion**	***urubu***<br>***vulture***
560.5	**ácido úrico aumentado**<br>**elevated uric acid**	dor no hálux<br>hallux pain	articulação inchada<br>swollen joint	***gota***<br>***goiter***
570	animal quadrúpede<br>quadruped animal	animal selvagem<br>wild animal	**juba exuberante**<br>**exuberant mane**	***leão***<br>***lion***
579.5	+	+	+	
589	vive na areia<br>lives in sand	anda de lado<br>walks sideways	**duas garras**<br>**two pincers**	***caranguejo***<br>***crab***
598.5	idoso<br>elderly	dores articulares<br>joint pains	**osteófitos**<br>**osteophytes**	***osteoartrite***<br>***osteoarthritis***
608	+	+	+	
617.5	**dispneia de decúbito**<br>**decubitus dyspnea**	cardiomegalia<br>cardiomegaly	edema pretibial<br>pretibial edema	***insuficiência cardíaca congestiva***<br>***congestive heart failure***
627	+	+	+	
636.5	teclado plástico<br>plastic keyboard	monitor de vídeo<br>video monitor	**disco rígido**<br>**hard drive**	***computador***<br>***computer***
646	**fabrica teia**<br>**builds webs**	produz veneno<br>produces venom	patas finas e longas<br>long and slender legs	***aranha***<br>***spider***
655.5	**dor na fossa ilíaca direita**<br>**right lower quadrant pain**	leucocitose<br>leucocytosis	obstipação<br>obstipation	***apendicite***<br>***appendicitis***
665	**sinalização de trânsito**<br>**traffic signaling**	luzes vermelha, amarela, verde<br>red, yellow, green lights	cruzamento de ruas<br>crossroads	***semáforo***<br>***traffic light***
674.5	+	+	+	

5- Set of stimuli for Experiment 2

To help the visualization of the task, stimuli are placed in the order in which they were presented to participants using one of the sets employed in the experimental protocol, including training stimuli. In the first column of the table is the temporal order of the presentation of stimuli in seconds. Null events are marked with crosses

Training

**temporal sequence**	**task**	**stimulus**	***a plausible response***
0	TREATMENT	dermatomicose nos pés<br>foot dermatomycosis	***antifúngico***<br>***antifungal***
6.5	+	+	
13	DIAGNOSIS	dor precordial recorrente<br>recurring precordial pain	***angina***<br>***angina***
19.5	DIAGNOSIS	hiperglicemia<br>hyperglycemia	***diabetes***<br>***diabetes***
26	+	+	
32.5	TREATMENT	gota<br>goiter	***alopurinol***<br>***allopurinol***
39	DIAGNOSIS	hematúria<br>hematuria	***nefrolitíase***<br>***nephrolithiasis***
45.5	+	+	
52	TREATMENT	gripe<br>influenza	***sintomárico***<br>***symptomatic***
58.5	+	+	
65	TREATMENT	anemia ferropriva<br>iron deficiency anaemia	***ferro***<br>***iron***
71.5	DIAGNOSIS	polidipsia<br>polydypsia	***diabetes***<br>***diabetes***
78	TREATMENT	sífilis primária<br>primary syphillis	***penicilina***<br>***penicillin***
84.5	+	+	
91	TREATMENT	pneumotórax hipertensivo<br>hypertensive pneumothorax	***descompressão***<br>***decompression***
97.5	DIAGNOSIS	amilase aumentada<br>elevated amylase	***pancreatite***<br>***pancreatitis***
104	+	+	
110.5	DIAGNOSIS	FAN postivo<br>ANF present	***lupus***<br>***lupus***
117	+	+	
123.5	TREATMENT	doença de Parkinson<br>Parkinson's disease	***levodopa***<br>***levodopa***
130	DIAGNOSIS	creatinina elevada<br>elevated creatinine	***insuficiência renal***<br>***renal insufficiency***
136.5	+	+	
143	TREATMENT	gastrite<br>gastritis	***omeprazol***<br>***omeprazole***
149.5	DIAGNOSIS	rinorréia purulenta<br>purulent rhinorrhea	***sinusite***<br>***sinusitis***

First session

temporal sequence	task	stimulus	*a plausible response*
0	DIAGNOSIS	febre terçã<br>tertian fever	***malária***<br>***malaria***
6.5	+	+	
13	TREATMENT	hepatite A<br>hepatitis A	***sintomático***<br>***symptomatic***
19.5	DIAGNOSIS	PPD positivo<br>positive PPD	***tuberculose***<br>***tuberculosis***
26	+	+	
32.5	DIAGNOSIS	ideação suicida<br>suicidal thoughts	***transtorno depressivo***<br>***depressive disorder***
39	DIAGNOSIS	perda de memória progressiva<br>progressive memory loss	***demência***<br>***dementia***
45.5	+	+	
52	TREATMENT	apendicite aguda<br>acute appendicitis	***cirurgia***<br>***surgery***
58.5	+	+	
65	TREATMENT	ascaridíase<br>ascaridiasis	***albendazol***<br>***albendazole***
71.5	DIAGNOSIS	cardiomegalia<br>cardiomegaly	***insuficiência cardíaca***<br>***heart failure***
78	TREATMENT	sarampo<br>measles	***sintomático***<br>***symptomatic***
84.5	+	+	
91	DIAGNOSIS	dispnéia de decúbito<br>decubitus dyspnea	***ICC***<br>***CHD***
97.5	+	+	
104	DIAGNOSIS	condensação pulmonar<br>pulmonary condensation	***pneumonia***<br>***pneumonia***
110.5	TREATMENT	sinusite bilateral<br>bilateral sinusitis	***antibiótico***<br>***antibiotic***
117	+	+	
123.5	DIAGNOSIS	regurgitação<br>regurgitation	***refluxo***<br>***reflux***
130	+	+	
136.5	TREATMENT	candidíase oral<br>oral candidiasis	***Micostatin***<br>***Mycostatin***
143	DIAGNOSIS	desânimo<br>despondent	***transtorno depressivo***<br>***depressive disorder***
149.5	+	+	
156	TREATMENT	escarlatina<br>scarlet fever	***antibiótico***<br>***antibiotic***
162.5	DIAGNOSIS	dor em fossa ilíaca direita<br>right lower quadrant pain	***apendicite***<br>***appendicitis***
169	+	+	
175.5	DIAGNOSIS	otorreia<br>otorrhea	***otite***<br>***otitis***
182	+	+	
188.5	DIAGNOSIS	dor supra-púbica<br>suprapubic pain	***cistite***<br>***cystitis***
195	TREATMENT	giardíase<br>giardiasis	***metronidazol***<br>***metronidazole***
201.5	TREATMENT	SIDA<br>AIDS	***antiretroviral***<br>***antiretroviral***
208	+	+	
214.5	DIAGNOSIS	chiados<br>wheezes	***asma***<br>***asthma***
221	+	+	

227.5	TREATMENT	refluxo gastroesofágico<br>gastroesophageal reflux	***orientação alimentar<br>nutritional recommendations***
234	TREATMENT	colecistite aguda<br>acute cholecystitis	***cirurgia<br>surgery***
240.5	TREATMENT	abscesso de pele<br>skin abscess	***drenagem<br>drainage***
247	+	+	
253.5	DIAGNOSIS	congestão nasal<br>nasal congestion	***rinite<br>rhinitis***
260	TREATMENT	trombose venosa profunda<br>deep venous thrombosis	***anticoagulante<br>anticoagulant***
266.5	+	+	
273	DIAGNOSIS	dor no hálux<br>hallux pain	***gota<br>goiter***
279.5	DIAGNOSIS	medo súbito<br>sudden fear	***transtorno do pânico<br>panic disorder***
286	TREATMENT	tuberculose de pulmão<br>pulmonary tuberculosis	***rifampicina<br>rifampicin***
292.5	DIAGNOSIS	hiperemia conjuntival<br>conjunctival hyperemia	***conjuntivite<br>conjunctivitis***
299	+	+	
305.5	TREATMENT	linfoma<br>lymphoma	***quimioterapia<br>chemotherapy***
312	+	+	
318.5	TREATMENT	herpes zoster<br>herpes zoster	***aciclovir<br>acyclovir***
325	DIAGNOSIS	rinorreia clara<br>clear rhinorrhea	***rinite<br>rhinitis***
331.5	+	+	
338	DIAGNOSIS	amenorréia<br>amenorrhea	***gravidez<br>pregnancy***
344.5	+	+	
351	TREATMENT	hipercolesterolemia<br>hypercholesterolemia	***estatina<br>statin***
357.5	DIAGNOSIS	hemoglobina baixa<br>low hemoglobin	***anemia<br>anemia***
364	+	+	
370.5	DIAGNOSIS	icterícia<br>jaundice	***hepatite<br>hepatitis***
377	TREATMENT	osteoartrite<br>osteoarthritis	***anti-inflamatório<br>antiinflammatory***
383.5	TREATMENT	hérnia inguinal estrangulada<br>incarcerated inguinal hernia	***cirurgia<br>surgery***
390	DIAGNOSIS	dor de ouvido<br>earache	***otite<br>otitis***
396.5	+	+	
403	TREATMENT	síndrome do pânico<br>panic disorder	***antidepressivo<br>antidepressant***
409.5	+	+	
416	TREATMENT	teníase<br>taeniasis	***antihelmíntico<br>antihelmintic***
422.5	DIAGNOSIS	VDRL positivo<br>positive VDRL	***sifílis<br>syphillis***
429	TREATMENT	câncer de mama<br>breast cancer	***cirurgia<br>surgery***
435.5	+	+	
442	TREATMENT	caxumba<br>mumps	***sintomático<br>symptomatic***
448.5	TREATMENT	tenossinovite no antebraço<br>forearm tenosynovitis	***anti-inflamatório<br>antiinflammatory***
455	DIAGNOSIS	TSH diminuido<br>low TSH	***hipertireoidismo<br>hyperthyroidism***
461.5	+	+	

Second session

temporal sequence	task	stimulus	*a plausible response*
0	DIAGNOSIS	dor em hipocondrio direito<br>upper right quadrant pain	***colecistite***<br>***cholecystitis***
6.5	+	+	
13	DIAGNOSIS	parestesia em bota<br>stocking paresthesia	***neuropatia periférica***<br>***peripheral neuropathy***
19.5	+	+	
26	TREATMENT	hipertensão arterial<br>arterial hypertension	***antihipertensivos***<br>***antihypertensive***
32.5	DIAGNOSIS	prurido ocular<br>ocular pruritus	***conjuntivite***<br>***conjunctivitis***
39	DIAGNOSIS	troponina aumentada<br>elevated troponin	***infarto do miocárdio***<br>***myocardial infarction***
45.5	+	+	
52	TREATMENT	disfunção erétil<br>erectile dysfunction	***sildenafil***<br>***sildenafil***
58.5	+	+	
65	TREATMENT	abdôme agudo<br>acute abdomen	***cirurgia***<br>***surgery***
71.5	DIAGNOSIS	fator reumatóide<br>rheumatoid factor	***artrite reumatóide***<br>***rheumatoid arthritis***
78	+	+	
84.5	TREATMENT	pólipo de cólon<br>colonic polyp	***polipectomia***<br>***polypectomy***
91	DIAGNOSIS	eritema em borboleta<br>butterfly rash	***lupus***<br>***lupus***
97.5	TREATMENT	amigdalite bacteriana<br>bacterial amygdalitis	***antibiótico***<br>***antibiotic***
104	+	+	
110.5	DIAGNOSIS	osteófitos<br>osteophytes	***osteoartrose***<br>***osteoarthrosis***
117	DIAGNOSIS	TSH aumentado<br>elevated TSH	***hipotireoidismo***<br>***hypothyroidism***
123.5	+	+	
130	TREATMENT	endocardite bacteriana<br>bacterial endocarditis	***antibiótico***<br>***antibiotic***
136.5	DIAGNOSIS	ascite<br>ascites	***hipertensão portal***<br>***portal hypertension***
143	DIAGNOSIS	hemiparesia súbita<br>sudden hemiparesis	***AVC***<br>***stroke***
149.5	+	+	
156	TREATMENT	insuficiência mitral grave<br>severe mitral insufficiency	***cirurgia***<br>***surgery***
162.5	DIAGNOSIS	dor de garganta<br>throat pain	***amigdalite***<br>***amygdalitis***
169	+	+	
175.5	DIAGNOSIS	nódulo mamário<br>breast nodule	***câncer***<br>***cancer***
182	TREATMENT	pneumonia lobar<br>lobar pneumonia	***antibiótico***<br>***antibiotic***
188.5	+	+	
195	DIAGNOSIS	aumento de parótidas<br>increased parotids	***caxumba***<br>***mumps***
201.5	+	+	
208	TREATMENT	cistite aguda<br>acute cystitis	***antibiótico***<br>***antibiotic***
214.5	TREATMENT	úlcera péptica<br>peptic ulcer	***omeprazol***<br>***omeprazole***
221	+	+	
227.5	DIAGNOSIS	disuria<br>dysuria	***ITU***<br>***UTI***

234	+	+	
240.5	DIAGNOSIS	ácido urico aumentado<br>elevated uric acid	***gota***<br>***goiter***
247	TREATMENT	otite média supurada<br>purulent middle ear otitis	***antibiótico***<br>***antibiotic***
253.5	DIAGNOSIS	tremor nas mãos<br>shaking hands	***doença de Parkinson***<br>***Parkinson's disease***
260	+	+	
266.5	TREATMENT	febre tifóide<br>thyphoid fever	***antibiótico***<br>***antibiotic***
273	DIAGNOSIS	rigidez nucal<br>nuchal rigidity	***meningite***<br>***meningitis***
279.5	+	+	
286	DIAGNOSIS	palidez de mucosas<br>pale mucosa	***anemia***<br>***anemia***
292.5	TREATMENT	aneurisma de aorta abdominal<br>abdominal aortic aneurysm	***cirurgia***<br>***surgery***
299	DIAGNOSIS	caverna pulmonar<br>pulmonary cavern	***tuberculose***<br>***tuberculosis***
305.5	+	+	
312	TREATMENT	epilepsia<br>epilepsy	***anticonvulsivante***<br>***anticonvulsant***
318.5	DIAGNOSIS	beta HCG elevada<br>elevated beta HCG	***gravidez***<br>***pregnancy***
325	+	+	
331.5	DIAGNOSIS	cefaléia latejante<br>throbbing headache	***enxaqueca***<br>***migraine***
338	TREATMENT	cólica renal<br>renal colic	***morfina***<br>***morphine***
344.5	TREATMENT	asma<br>asthma	***Berotec***<br>***Berotec***
351	+	+	
357.5	TREATMENT	escabiose<br>scabies	***ivermectina***<br>***ivermectin***
364	DIAGNOSIS	variz esofágica<br>esophageal varices	***cirrose***<br>***cirrhosis***
370.5	+	+	
377	TREATMENT	depressão<br>depression	***antidepressivo***<br>***antidepressant***
383.5	TREATMENT	hipotireoidismo<br>hypothyroidism	***reposição hormonal***<br>***hormone replacement***
390	+	+	
396.5	TREATMENT	tromboembolismo pulmonar<br>pulmonary embolism	***anticoagulante***<br>***anticoagulant***
403	DIAGNOSIS	fogachos<br>hot flashes	***menopausa***<br>***menopause***
409.5	+	+	
416	TREATMENT	tricomoníase<br>trichomoniasis	***metronidazol***<br>***metronidazole***
422.5	+	+	
429	TREATMENT	impetigo<br>impetigo	***antibiótico***<br>***antibiotic***
435.5	TREATMENT	leucemia<br>leukemia	***quimioterapia***<br>***chemotherapy***
442	+	+	
448.5	DIAGNOSIS	retração de mamilo<br>nipple retraction	***câncer de mama***<br>***breast cancer***
455	TREATMENT	catarata bilateral<br>bilateral cataract	***cirurgia***<br>***surgery***
461.5	+	+	
